# Genetic Mapping and Evolutionary Analyses of the Black Grain Trait in Barley

**DOI:** 10.3389/fpls.2018.01921

**Published:** 2019-01-08

**Authors:** Zhoukai Long, Yong Jia, Cong Tan, Xiao-Qi Zhang, Tefera Angessa, Sue Broughton, Sharon Westcott, Fei Dai, Guoping Zhang, Dongfa Sun, Yanhao Xu, Chengdao Li

**Affiliations:** ^1^Hubei Collaborative Innovation Centre for Grain Industry, Yangtze University, Jingzhou, China; ^2^Western Barley Genetic Alliance, Murdoch University, Perth, WA, Australia; ^3^Department of Agriculture and Food, Government of Western Australia, South Perth, WA, Australia; ^4^College of Agriculture and Biotechnology, Zhejiang University, Hangzhou, China; ^5^College of Plant Science, Huazhong Agricultural University, Wuhan, China

**Keywords:** barley, black lemma and pericarp, genetic mapping, wild barley, evolution, environmental adaptation

## Abstract

Barley occupies the widest ecological area among the major cereal crops, thereby generating a high potential for adaptive genetic diversity against various environmental factors. Colored barley such as black grain barley has been suggested to result from environmental adaptation to biotic and abiotic stresses. Using one double haploid population (433 lines), plus three F5 recombinant inbred line (RIL) populations (1,009 lines), the black lemma and pericarp (*Blp*) gene was mapped between two Insertion/deletion (Indel) markers MC_1570156 and MC_162350 with a physical distance of 0.807 Mb, containing 21 annotated genes in the mapped interval. Whole-genome re-sequencing was performed on two Tibetan wild barley lines (X1 and W1) with black grain phenotype. The probable candidate genes for *Blp* were discussed based on gene functional annotation and gene sequence variation analyses. Thirteen polymorphic Indel markers covering the target genetic region were used to analyze 178 barley accessions including 49 black husk entries. Genotype-based clustering analyses showed that the black landraces of different geographical background may have evolved from a single origin. Our study represents a significant improvement on the genetic mapping of *Blp* and would facilitate future study on the characterization of the genetic basis underlying this interesting agronomic trait.

## Introduction

Climate change, varying environmental condition and limited natural resources such as water and land pose significant challenge for human to produce sufficient food to feed a growing population (Beddington et al., 2012). Cultivated barley (*Hordeum vulgare* subsp. *vulgare*), ranked as the fourth most important cereal crop after wheat, rice, and maize (USDA USDoA., 2017), has adapted to a broad spectrum of agricultural environments that differs in water availability, temperature, soil type, altitude etc (Zhang et al., 2012). Its wild progenitor *H. vulgare* subsp. *Spontaneum*, distributed in an extensive ecological range from the east-Mediterranean basin, North Africa to central Asia and the Tibetan highland of China (Nevo, 2012; Zohary et al., 2012), is rich in genetic diversity (Russell et al., 2014) and continues to provide a valuable source of alleles to cope with changing environments (Nevo et al., 2012). Both cultivated barley and wild barley are diploid, self-pollinated, and fully interfertile (Zohary et al., 2012). Due to its broad geographic distribution and great potential of genetic diversity, barley has become an excellent model to investigate crop's adaption to various environmental conditions.

The grain color of barley is an important crop trait under agricultural selection. Colored barley is rich in phenolic acids and anthocyanin (Abdel-Aal et al., 2006, 2012). These flavonoid compounds have important free radical scavenging capacity (Abdel-Aal et al., 2012), thereby making colored barley a popular healthy food. Mature barley grain may develop different colors (yellow, purple, red, blue, black, and gray) due to different pigmentations (Hua et al., 2013). The blue barley is due to the coloration in the aleurone layer while the red and purple colors occur in the pericarp (Harlan, 1914; Lundqvist et al., 1997). In both cases, pigmentations are caused by the accumulation of anthocyanins. Delphinidin-3-glucoside dominates the anthocyanins in the blue barley while caynidin-3-glucoside is the most abundant in purple and red (Kim et al., 2007; Siebenhandl et al., 2007). The yellow barley is due to the accumulation of proanthocyanidins in the testa layer (Aastrup et al., 1984). Unlike the other colored barleys, the black and gray color has been attributed to the accumulation of phytomelanins in the lemma and pericarp (Harlan, 1914; Lundqvist et al., 1997). Notably, significant amount of anthocyanin, mainly delphinidin-3-glucoside (Kim et al., 2007; Siebenhandl et al., 2007), have also been found in in the middlings of the black barley kernel (Siebenhandl et al., 2007). Compared to other colored barley, black barley is environmentally stable and has exceptional dietary benefits. It contains a relatively higher level of anthocyanin and lignin (Choo et al., 2005), and has been shown to be more resistant to *Fusarium* disease (Choo et al., 2015). The genetic locus (*Blp*) controlling this trait has been mapped to chromosome 1HL (Costa et al., 2001; Bungartz et al., 2016; Shoeva et al., 2016; Jia et al., 2017).

Despite the well-characterized phytochemistry basis, the biological explanation on why barley grain develops various colors is primarily lagging behind. Colored barley is rich in anthocyanin (Abdel-Aal et al., 2006; Kim et al., 2007). Anthocyanin accumulation in plants has been suggested to play a role in tolerance to diverse environmental stressors such as drought, temperature, UV and heavy metals, as well as resistance to herbivore and pathogens (Winkel-Shirley, 2001; Gould, 2004). Colored barley accounts for over 68% of the wild barley accessions in Tibet region (Choo, 2002), which is well-known for its harsh environmental conditions, including high-altitude, UV exposure, low temperature etc. At an altitude of 4,000 m and above, all barley have dark-colored (blue, red, purple, black etc) kernels (Choo, 2002). This suggests that the diverse barley grain colors may be plants' response to environmental stressors and may have evolved as a result of environmental adaptation. Black barley has been found to have pronounced drought tolerance compared to normal white barley (Yasseen and Almaamari, 1995). Previous study on colored barley, including black, purple, blue, and yellow, have shown that colored barley is rich in genetic diversity and have a complex evolutionary relationship (Hua et al., 2015). Genetic and evolutionary analyses of grain color are needed to understand its potential interaction with environmental conditions.

In this study, we aim to investigate the genetic basis and evolutionary characteristics of the black lemma and pericarp (*Blp*) trait in barley. Four populations (1 DH and 3 F5 RIL, total 1,442 lines) were used to map the *Blp* gene. Whole-genome re-sequencing of two Tibetan black lines were performed. Genetic variation analyses and natural sweep analyses of the target genetic region were carried out. The most likely candidate gene was discussed. In addition, 13 polymorphic markers close to the *Blp* gene were used to genotype 178 barley lines (49 black) of diverse ecological backgrounds. Genotype-based clustering analyses were performed. The results significantly contribute to our understanding of the genetic basis and evolutionary characteristics of the black coloration in barley.

## Results

### Inheritance and Morphology Analyses of the Black Lemma and Pericarp Trait in Barley

**Figure 1:**
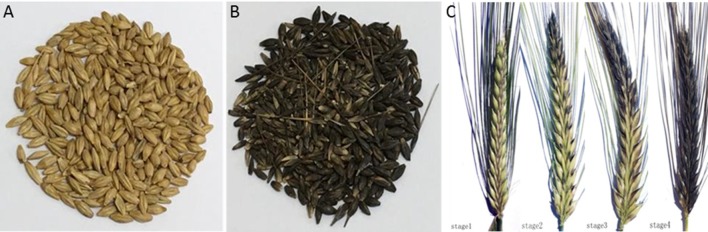
Morphological observations of parental lines. **(A)** Hindmarsh; **(B)** W1; **(C)** Color changes of the black lemma and pericarp trait in developing barley spike (W1/Hindmarsh DH line, 5 days interval).

Parent lines Hindmarsh and W1 display yellow and black lemma/pericarp, respectively (Figures 1A,B). F1 seeds were obtained by crossing W1 to Hindmarsh. Since the black lemma/pericarp trait displayed maternal effects, the husk and seed color phenotype of F1 population was recorded by examining the seed color the mature F2 seeds. The results showed that all F2 seeds display black color (data not shown). In addition, 433 DH lines were also developed for W1 x Hindmarsh. In the DH population, there are 223 black lines and 210 yellow lines. Chi-square test (*X*^2^ = 0.390; *p* = 0.5321) showed that the results fit a segregation ratio of 1:1, indicating that the *Blp* trait was controlled by a single gene. The developmental changes of the *Blp* trait were recorded at four serial stages (5 days interval). As shown in Figure 1C, the black coloration starts from the top of the spike, extends to the middle later and to the whole spike in the end.

### Preliminary and Fine Mapping of the *Blp* Gene Based on SNP and Indel Markers

**Figure 2:**
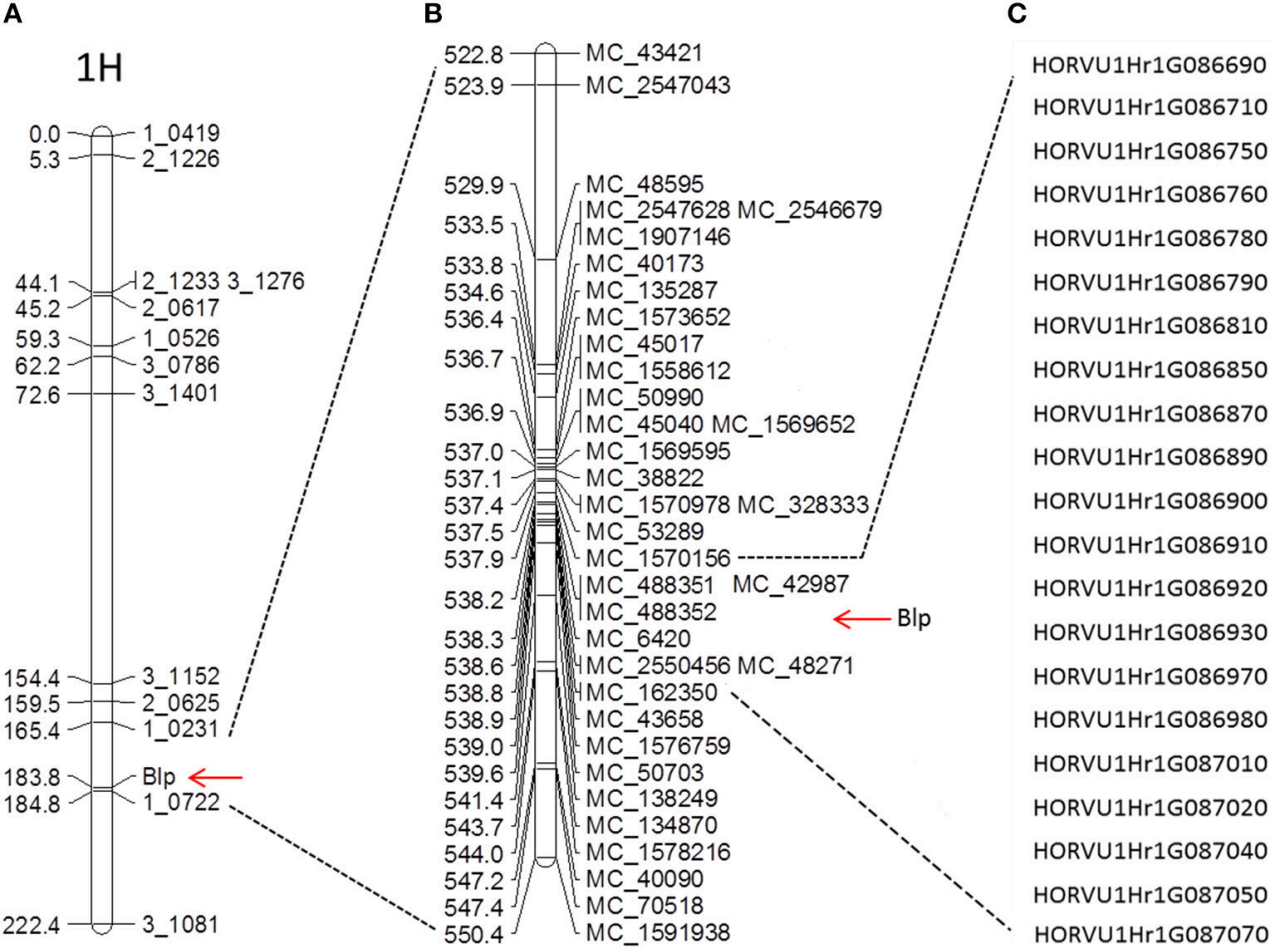
Marker linkage analysis and fine genetic mapping of the *Blp* gene. **(A)** Preliminary SNP marker linkage map; **(B)** Fine genetic mapping using newly designed indel markers; **(C)** Predicted candidate genes between markers MC_1570156 and MC_ 162350.

A total of 384 single nucleotide polymorphism (SNP) markers were selected from the Golden Gate barley OPA snp marker panel (Illumina, California, United States) to test polymorphism between Hindmarsh and W1. Ninety five SNP markers evenly distributed across the whole genome were used to analyze the 188 DH lines (88 yellow and 100 black). The resulting data was input to JohinMap 4.0 (Van Ooijen, 2006) to generate a preliminary linkage group map (Additional file 1). Seven chromosome linkage groups were identified, corresponding to chromosome 1–7H. The *Blp* gene was positioned between 1_0231 and 1_0722 on chromosome 1H (Figure 2A). The closest marker to the *Blp* gene is 1_0722, with a genetic distance of 1.0 cM. Due to the close distance between *Blp* and 1_0722, the genetic regions around 10 Mb upstream and downstream of 1_0722 were selected for fine mapping (Figure 2B). We designed 71 pairs of InDel markers in the target region based on the genome sequences of Morex, Bark, and Bowman in the Barley IPK database (http://webblast.ipk-gatersleben.de/barley/). Of these, 25 markers display polymorphism between the parent lines Hindmarsh and W1 (Table 1). These markers were used to analyze the whole DH population (433 lines). As a result, the *Blp* gene was mapped between marker MC_48595 (529.88 Mb) and MC_50703 (539.71 Mb). No recombination line could be found between MC_48595 and MC_50703 in the DH population. To further map the *Blp* gene, another 59 indel markers was designed between MC_48595 and MC_50703. Firstly, a F5 RIL population TH1 (234 black, 146 yellow) was used to screen for recombinant lines between MC_48595 and MC_50703 (Additional file 2). Polymorphism was tested using the Bulked Segregant Analysis (BSA) method by combining DNA from 4 yellow and 4 black lines. This led to the identification of 11 polymorphic markers (Table 1). These markers were then used to analyze the other two F5 RIL populations TH2 (152 black, 161 yellow) and TH4 (147 black, 169 yellow) (Additional file 2). The results positioned the *Blp* gene between MC_1570156 (537.86 Mb) and MC_162350 (538.66 Mb), with a physical interval of 0.807 Mb.

**Table 1:** List of newly designed Indel markers used for fine mapping.

**ID**	**Forward**	**Reverse**	**Position (forward)**	**PCR size**	**Alleles**	**Tm (**°**C)**
MC_43421	GTCAAGACCCGGCCGATATA	TTGTCTGTCTGGCTGTGCTC	522847678	91		60
**MC_2547043**	**CGAACCAAACGCCACAACAT**	**ACGGTTCTTTAGGCGCCAAA**	**523882337**	**102**	**2**	60
MC_48595	TAGAGATGCTTGATCCGCCG	CCAAGGACACTTAGCGGAGG	529880207	101		60
**MC_2546679**	**TCCCTGTGTGAACACTCTGC**	**AACCAGCTGATTTTACGCGC**	**533308880**	**88**	**2**	60
MC_1907146	ACGATGATGGTCCCTACCCT	TCGAACAGAGGCAAAGGGAC	533324365	88		60
MC_2547628	GTCAGATGGCAGCCAGAGAA	TGTCGGAAGAACTCACACGG	533335063	110		60
MC_40173	AAAACCGCGTTGAATCCTGC	GACCGGGGACACTGTTCATG	533872376	120		60
MC_135287	CATGATCTCCTGCGCCTTGA	AAGTGGCATGCTCGTCAGAA	534618575	156		60
MC_1573652	CCGTTTGTGAGCATATCTGTCG	ACGCTCACAAATGGACAGCA	536421753	111		60
**MC_45017**	**ATGAGAAAGTTGGGGTGCAC**	**ACAGTATCCAACATGGGTGTGT**	**536616456**	**102**	**4**	60
MC_1558612	TCGCCAAGTCTCTATGTTCCG	TTGGGGCTGCAAGTAGATGG	536783684	131		60
MC_50990	CCTGCGATTGGATGATGGGA	GGTCCCTTGTCTGCTCAGTC	536887917	130		60
MC_1569595	CCATGCTCGCCTCCTCCT	GTCGAGTCCACTGCCTCG	536946178	92		60
MC_1569652	GGCTAATGTCAGTAATTGTTGCGT	ACGTCACGAGATTGTATTGTGAC	536975893	93		60
MC_45040	CACGGTCGTGCCTGTTAGAA	AGAGCAAGTCCGTTTTCCGT	536985141	292		60
MC_38822	ATCTACTGACGCACCTGACG	GGTTCGCTGATGGTCTGGAT	537080913	135		60
MC_328333	GCCGGCTGTGCTCTTAAAGT	TGGTCTTAGACATAGGAAAAAGTTGT	537359466	141		60
MC_1570978	CTTGGCAGGAGAAATGCTGC	CGCTGCTTGAGACGGTAGT	537365649	136		60
**MC_53289**	**GAGCGAGTCTGTGCTTCCAT**	**CCCACTTGACACCTTCCTCC**	**537506795**	**128**	**3**	60
**MC_1570156**	**CGCCTCTACATACTTCAAGGGT**	**CTGTGGCTACTGCAAAGTTTGT**	**537855051**	**119**	**2**	60
**MC_42987**	**CATCGGATCACATCGCATCG**	**AAGTGGCCAGCAACGAAATG**	**538130565**	**148**	**2**	60
**MC_488351**	**TACCGTTGCATCGTTGGACA**	**CACAAATATGCCAGCGACGG**	**538131236**	**173**	**2**	60
**MC_488352**	**GGAAAGTGCACTGGACAACG**	**GCTCACGTGTGCTTGAACAG**	**538135118**	**145**	**3**	60
MC_6420	GATCAACCCCTTGGAGAACTTG	AATTGTCCTCAACCATCATCAAGGC	538481680	423		60
**MC_2550456**	**GGCCTCAAGAACCTTT**	**ACAGACGAGACAAAAAGAGTCA**	**538583950**	**95**	**2**	58
**MC_162350**	**GCAAAATTTGGCAGCCACAG**	**AAATTTGTGCTGGGTGGTGG**	**538661850**	**89**	**2**	60
**MC_48271**	**CCTGTCCTTCGGGTCCCTAT**	**TGCAACGGTAGGCAATTTAT**	**538715652**	**127**	**4**	62
MC_43658	TCCTCGTCCTCGTCCTCTTC	AGCCACAGATAAGCAGGCTG	538889945	135		60
**MC_1576759**	**TGTGCAGTGATATGAGGTGACA**	**TGCTCACCAGACCAGTTTTGT**	**539107144**	**116**	**3**	60
MC_50703	TAACTGCCCATTGCTGATGC	TGCCCTATTTGACAGCCTCTG	539707746	148		60
MC_138249	CGTACGTACGTTGGTCTCGG	TAGCATATGCAGCGGTGGAG	541493652	107		60
MC_134870	GTTTTTGTGGGGTTTCCGCT	CTGGAGCAAATCGGATGGGT	543761399	141		60
**MC_1578216**	**TTAACGCGCCCCTTGGAG**	**TGCTGCAGCGATGATGGAG**	**544020811**	**131**	**2**	60
MC_40090	TCGCCCTCGATTAAAACCGA	TTCAAATCCATCCCGAACCA	547267833	121		60
MC_70518	CAGAATGAACGGCGAGGGTA	AGAAGCAGATGTTGTCGCCA	547450494	129		60
MC_1591938	CCGCCGAGGATGATAATCGT	TGTTGTGAGCAAACGGGAAC	550308237	97		60

*The markers selected for the clustering analyses and their corresponding number of alleles are highlighted in bold. “Tm” refers the annealing temperature*.

### Genetic Variation Analyses Based on Genome Re-sequencing of Two Black Barley Lines

To investigate the genetic variation that may have contributed to the *Blp* trait in barley, whole genome re-sequencing was performed using two black Tibetan barley lines (W1, X1) and two yellow cultivated barley cultivars (AC_Metcalfe, Baudin). The sequence information for the identified chromosomal region (0.807 Mb) was extracted from the whole genome re-sequencing data. Sequence comparison with the reference Morex genome (Mascher et al., 2017) revealed significant variation in the target genetic region (Additional file 3). The common unique mutations identified between W1/X1 and AC_Metcalfe/Baudin and their relative position related to the annotated genes were summarized in Table 2. In total, 16 Indel and 89 SNP mutations in the transcriptional regions were identified between W1/X1 and AC_Metcalfe/Baudin. According to the most recent barley genome annotation results, 14 out of the 16 indel mutations were found in the 3' or 5' untranslated region, while the other 2 indels occurred in the coding region causing a in-frame shift and a frame-shift respectively in HORVU1Hr1G086920 and HORVU1Hr1G086780 respectively (Table 2). In addition, another 17 indel mutations were identified in the gene upstream or gene downstream region (Additional file 3). These mutations may cause the alteration of the transcription of the corresponding gene. The other indels identified were located in the inter-genic region and may have little effect on the gene transcription (Additional file 3). Most of the SNP mutations occurred in the inter-genic, gene upstream or gene downstream region and may have little significance concerning the *Blp* trait. Regarding the SNPs identified in the transcriptional region, 38 SNPs were found in the 3' or 5' UTR region. In addition, 28 SNPs in the coding region are predicted to cause amino acid mutation (Table 2). Further examination is needed to evaluate the potential effect of the indel and SNP mutations on gene function.

**Table 2:** List of the candidate genes and the summary of unique mutations between W1/X1 (black) and AC_me/ BD (yellow).

**Gene ID**	**Functional annotations**	**Pfam number**	**3' UTR (SNP/indel)**	**5' UTR (SNP/indel)**	**Inframe indel**	**Frameshift indel**	**Missense SNP**	**Synonymous SNP**	**Splice region SNP**	**Sum**
HORVU1Hr1G086690	Ubiquitin-conjugating enzyme 35	PF00179	–	–	–	–	–	–	–	0
HORVU1Hr1G086710	Gibberellin 2-oxidase	PF03171; PF14226	–	–	–	–	–	–	–	0
HORVU1Hr1G086750	Chlororespiratory reduction31	PF11623	–	–	–	–	–	–	–	0
HORVU1Hr1G086760	3-ketoacyl-CoA synthase 5	PF08392; PF08541	1/0	–	–	–	–	–	–	1
HORVU1Hr1G086780	Undescribed protein	none	3/0	–	–	1	3	1	–	8
HORVU1Hr1G086790	Undescribed protein	none	–	0/1	–	–	1	–	–	2
HORVU1Hr1G086810	Gibberellin 2-oxidase	PF03171; PF14226	–	–	–	–	–	2	–	2
HORVU1Hr1G086850	Undescribed protein	PF08186	–	–	–	–	–	–	–	0
HORVU1Hr1G086870	Undescribed protein	none	–	1/0	–	–	–	–	–	2
HORVU1Hr1G086890	Beta-fructofuranosidase, insoluble isoenzyme 1	PF00251; PF08244	2/1	–	–	–	4	2	–	9
HORVU1Hr1G086900	Calmodulin like 43	PF13499	–	–	–	–	1	–	–	1
HORVU1Hr1G086910	Undescribed protein	none	6/1	1/0	–	–	7	–	2	17
HORVU1Hr1G086920	Cytochrome P450 superfamily protein	PF00067	–	–	1	–	3	–	–	4
HORVU1Hr1G086930	Undescribed protein	PF07223	0/1	3/1	–	–	–	2	–	7
HORVU1Hr1G086970	Undescribed protein	none	–	0	–	–	3	1	–	4
HORVU1Hr1G086980	3-deoxy-manno-octulosonate cytidylyltransferase	PF02348	–	9/1	–	–	3	1	–	14
HORVU1Hr1G087010	Purple acid phosphatase 27	PF00149; PF14008; PF16656	1/0	9/3	–	–	4	4	4	25
HORVU1Hr1G087020	Calcium-dependent protein kinase	none	–	–	–	–	–	–	–	0
HORVU1Hr1G087040	Chaperone protein DnaJ	none	–	–	–	–	–	–	–	0
HORVU1Hr1G087050	Receptor-like protein kinase 4	PF00069; PF07714	–	–	–	–	–	–	–	0
HORVU1Hr1G087070	DnaJ homolog subfamily B member 13	PF00226; PF01556	2/5	–	–	–	–	3	1	11

### Gene Prediction in the Target Genetic Region

The *Blp* gene was mapped between indel markers MC_1570156 and MC_ 162350 in the present study. According to the most recent barley genome annotation, there are a total of 21 candidate genes located in this region (Figure 2C and Table 2). Of these genes, 6 genes were annotated with unknown function. The rest were predicted to encode for protein from diverse families (Table 2). Notably, 7 of the 21 candidate genes display no sequence variation between W1/X1 and AC_Metcalfe/Baudin (Table 2) and thereby may be excluded for the search of the candidate *Blp* gene. We searched the amino acid sequences encoded by the other 14 candidate genes in the Uniprot database (http://www.uniprot.org/), no homolog to currently known enzymes or transcription factors in the anthocyanin pathway was found. Previous studies indicated that the black pigmentation in barley is caused by the accumulation of phytomelanins or its co-pigmentation with anthocyanin in the lemma and pericarp (Mullick et al., 1958; Shoeva et al., 2016). It is reasonable to speculate that the *Blp* gene may be directly related to phytomelanins production. Based on protein homology search and functional prediction, HORVU1Hr1G087010, encoding for a putative purple acid phosphatase (PAP), may serve as a candidate gene responsible for the black pigmentation in barley lemma and pericarp. Functional domain analyses of HORVU1Hr1G087010 protein indicated that it is a metaloenzyme with two metal-binding sites. All known proteins in the PAP family are N-terminal glycosylated, a typical character for secreted plant enzymes (Olczak et al., 2003). The pigmentation may involve the secretion of PAP outside the cell and its iron-binding and reactive oxygen species generation function. Sequence variation analyses showed that HORVU1Hr1G087010 has the highest number of mutations among the candidate genes in the target genetic region. Most of these mutations (12) occur in the 5 prime UTR regions, while 4 mutations that cause amino acid changes were also identified for HORVU1Hr1G087010 (Table 2). In addition, 4 SNPs in the splice region were also identified between black and white barley. The other candidate genes displaying significant mutations in the 5 or 3 prime UTR regions include HORVU1Hr1G086970 (unknown function), HORVU1Hr1G086980 (3-deoxy-manno-octulosonate cytidylyltransferase) and HORVU1Hr1G087070 (DnaJ homolog subfamily B member 13). Interestingly, one in-frame indel and one frame-shift indel were identified for HORVU1Hr1G086920 (cytochrome P450 superfamily protein) and HORVU1Hr1G086780 (unknown protein) respectively (Table 2).

### Genotyping-Based Clustering Analyses of the Target Genetic Region in Barley

**Figure 3:**
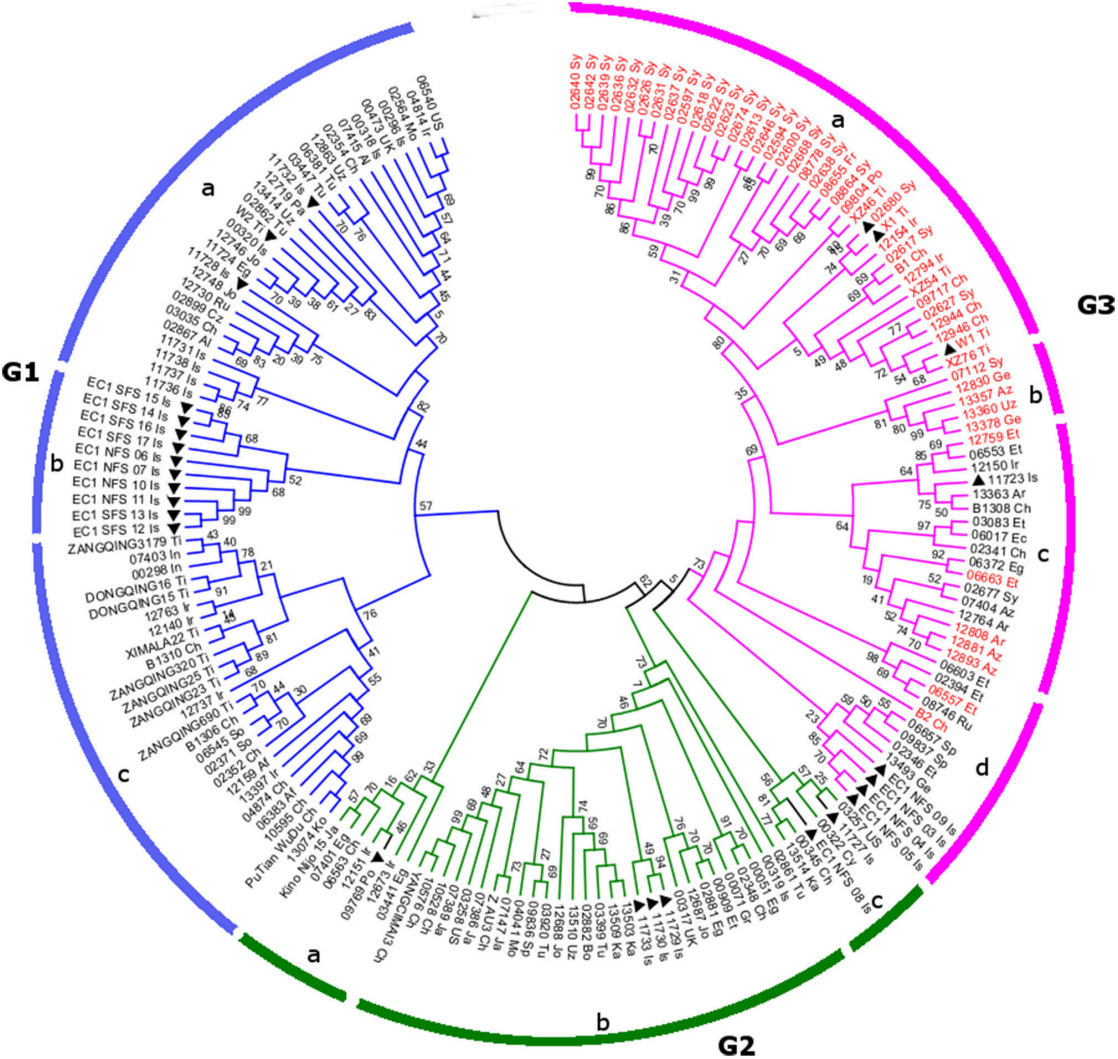
The phylogenetic tree displaying the clustering pattern of 178 barley accessions. The unrooted phylogenetic tree was inferred using the Neighbor Joining method based on the genotype data of 13 indel markers surrounding the target *Blp* gene. Barley accessions with black lemma and pericarp were annotated with red taxa color while the rest in black color. The last two letters of the taxa name represent the geographical location (see Additional file 4 for details). Wild barley was marked with solid triangle. The phylogenetic tree is divided into three major groups G1–3, which could be further separated into different subclades a–d.

In order to explore the evolutionary origin of the *Blp* gene in barley, indel markers were designed in the genetic region surrounding the identified target region. Thirteen polymorphic markers (Table 2) were identified. These markers were applied to 178 selected barley lines with diverse genetic background, which originates from different geographical locations. These lines include 49 accessions displaying the black husk trait (Additional file 4). Genotyping results showed that 9 out of the 13 markers have 3 alleles while the rest have 4 alleles (Table 2, Additional file 4). The representative gel pictures for each marker could be found in Additional file 5. An unrooted phylogenetic tree displaying the clustering pattern was obtained using the Neighbor Joining method. As shown in Figure 3, the selected barley accessions are divided into three major groups: G1–G3. All of these groups encompass barley accessions from diverse ecological backgrounds and displayed a geography-based clustering pattern, suggesting the clustering analysis results in the present study are reliable. G1 can be further divided into 3 subclades (G1-a, G1-b, and G1-c). G1-a mainly covers those accessions originated from the Near East Fertile Crescent and West Asia, while G1-b represents the wild barley accessions from Israel. In contrast, G1-c corresponds to barley originated from the Tibet and East Asia regions collectively. Regarding the barley accession in the 3 major subclades in G2 (Figure 3: G2-a, G2-b, and G2-c), there are some inter-group overlapping of their geographic locations. In particular, barley accessions collected from China, Egypt, and Israel can found in more than one subclade G2-a, G2-b, and G2-c. Despite this, a clear distinction between barley collected from the Near East Fertile Crescent and East Asia could still be identified in subclade G2-b. No Tibetan barley accession was found in G2. Interestingly, all the *Blp* barley included in the present study (49 accessions) are present in G3. Based on the bootstrapping value, G3 could separate into four putative subclades G3-a, G3-b, G3-c and G3-d. G3-a mainly covers barley accessions originated from Syria, China, and Tibet, with a couple of exceptional accessions from Portugal and France. G-b presents a small group of barley accessions from Western Asia. Notably, all members in G3-a and G3-b are black colored barley. In contrast to G3-a and G3-b, G3-c and G3-d display a mixture of black and yellow barley. These barley accessions mainly come from the Near East Fertile Crescent and Western Asia. Overall, the *Blp* barley accessions tend to group together, despite of their diverse geographic origins.

## Discussion

The *Blp* trait in barley has recently attracted great research interests (Bungartz et al., 2016; Shoeva et al., 2016; Jia et al., 2017), due to its potential as healthy food and its distinct pigmentation mechanism from other colored grains. In the present study, we fine mapped the *Blp* locus to a critical interval between two Indel markers MC_1570156 and MC_ 162350 on chromosome 1H. With the utilization of multiple populations with different genetic backgrounds, we confirmed previous reports that the *Blp* trait is controlled by a single dominant locus on chromosome 1HL. This location is consistent with the *Blp* locus reported by Costa et al. (2001), Bungartz et al. (2016), and Shoeva et al. (2016). Most recently, with the utilization of bulked segregant analysis (BSA) method, Jia et al. (2017) has mapped *Blp* to a genetic interval of 1.66 Mb on chromosome 1H. However, little attention has been paid toward the potential genetic and biochemistry basis underlying the *Blp* trait. The current study fine mapped *Blp* to an interval of only 0.807 Mb, which overlaps with that reported by Jia et al. (2017) and represents a significant improvement on the genetic mapping. Our study has been significantly facilitated by the most recent barley genome assembly (Mascher et al., 2017), which predicts only 21 annotated candidate genes in the mapped region. Based on the re-sequencing results of two black grain lines, the results presented here enable us to get even closer to uncover the molecular mechanism of the black colored grains and its environmental association in barley and other plants.

The pigmentations in most colored barley, such as yellow, red, blue and purple, are due to the production of flavonoid compounds (Abdel-Aal et al., 2006; Siebenhandl et al., 2007). As such, the candidate genes underlying these colorations would most likely regulate or participate in the flavonoid biosynthesis reactions. The *Pre2* gene responsible for the purple lemma and pericarp trait in barley has been identified to be a basic Helix-Loop-Helix transcription factor which regulates the anthocyanin synthesis pathway (Cockram et al., 2010). In addition to barley, many seed color genes cloned from maize (Carey et al., 2004), *Arabidopsis* (Nesi et al., 2000, 2002; Kitamura et al., 2004), rice (Furukawa et al., 2007), and rapeseed (Zhou et al., 2016) also corresponds to enzymes and transcription factors that are related to flavonoid biosynthesis. However, unlike other colored barley, the black coloration is caused by phytomelanin or its co-pigmentation with anthocyanin (Harlan, 1914; Lundqvist et al., 1997). The production of anthocyanin in black barley seems only an accompanying process. This hypothesis is supported by a recent study which showed that the specific transcriptional regulation of the flavonoid biosynthesis pathway genes is not detected in black barley (Shoeva et al., 2016). Indeed, our results revealed that no structural gene or transcription factor gene in the flavonoid biosynthesis pathway was predicted in our identified target genetic region (Table 2). Therefore, we focused on the phytomelanin metabolic pathway in the search of the candidate *Blp* gene.

Phytomelanin is a mechanically-hard, water-insoluble, brown-to-black colored resistant layer commonly found in the pericarp of some *Eupatorieae* and *Heliantheae* plants (Pandey et al., 1989; Pandey and Singh, 1994; Pandey and Dhakal, 2001). Anatomy analyses indicates that phytomelanin in *Clibadium, Desmanthodium*, and Ichthyo is non-cellular organic mass accumulated in the space between the fibrous layer and the hypodermal cell layers (Putt, 1944; Rogers et al., 1982). Polymerization occurs outside the cell walls of the inner hypodermal cells (Pandey and Dhakal, 2001; Tadesse and Crawford, 2014). Therefore, the candidate genes annotated in the target genetic region were scrutinized for those encoding for proteins that may be secreted outside the cell. This led to the identification of HORVU1Hr1G087010, which encodes for a putative PAP protein. Functional domain analysis suggests that HORVU1Hr1G087010 encoded protein is a metaloenzyme and contains two meta-binding sites (one iron-binding, the other iron or Zinc, Cu binding). The active enzyme of PAP has been suggested to be able to generate reactive oxygen species (Olczak et al., 2003), which may be essential for the phenolic acid oxidation process during phytomelanin synthesis. All known PAPs are N-terminal glycosylated, a typical character for secreted enzymes (Olczak et al., 2003). Sequence comparison of black barley lines with reference genome revealed 4 non-synonymous mutations in the coding region of HORVU1Hr1G087010 and 12 mutations in the 5 prime UTR region (Additional file 3), which may have altered the gene transcription, thereby contributing to the black coloration. However, other candidate genes couldn't be excluded until further fine mapping and functional verification are performed.

In addition to the exceptional health benefit, black barley also has important biological significance. Earlier study has indicated black barley is significantly less affected by *Fusarium* disease (Choo et al., 2015), which has been suggested to be related to the presence of flavonoid pigments and a relatively high level of lignin (Choo et al., 2005). It's very common to see plant to produce additional pigments under various biotic or abiotic environmental stresses (strong light, temperature, UV, drought, heavy metal, and insect, pathogen et al) (Chalker-Scott, 1999; Gould, 2004). Barley is a resilient crop that is distributed across an extensive ecological environment. The pigmentation in black barley as well as other colored barley may confer additional advantages for the plant to cope with marginal subsistence environments. Notably, over 68% of Tibetan wild barley accessions are colored barley. At an altitude higher than 4,000 m, all barley accessions have colored kernels (Choo, 2002). In our study, we observed that the black coloration emerged from the top of the spike and eventually extended to the base of the spike (Figure 1C), which suggests the pigmentation may be associated with light induction. A potential correlation between barley coloration and environmental adaptation may be proposed. In addition to Tibetan region, black barley has also been found in many other geographic locations. We identified 13 polymorphic markers near the target genetic region and used these markers to analyze 178 barley accessions. Genotype clustering analyses in the present study reveal an overall geographical-based pattern (Figure 3), which lends support to the environmental adaptation of black barley at the genetic level. Barley accessions under diverse ecological conditions tend to accumulate geography-specific mutation. In the present clustering analysis, barley collected from the West (Near East Fertile Crescent, Mediterranean-basin and Europe) can be clearly distinguished from those from the East (Tibet and East Asia). This is consistent with the report that Tibet acts as an independent evolutionary origin for the domesticated barley in East Asia (Dai et al., 2012; Ren et al., 2013). Notably, all the black barley accessions, spanning those from the West and the East, tend to cluster into a single clade in the present study. This indicates that the *Blp* trait is relatively conserved and may have evolved from a single origin that exists before the split between the wild barleys in the Near East and those in Tibet. This is supported by the observation that black barley from Tibet and East Asia form a separate clade from the rest black barley. Thirteen polymorphic markers were identified near the target *Blp* gene, covering a genetic region of around 10 Mb. The allele number identified for each marker is relatively low considering the diverse barley accessions analyzed (Table 1). In a previous study (Hua et al., 2015), SSR markers were used to analyze the genetic diversity of colored barley. The analysis revealed rich genetic diversity and a complex evolutionary relationship in the colored barley populations. The discrepancy might be because their study used SSR markers distributed across the whole barley genome while our study focused on the genetic region covering the *Blp* locus. Our analysis is more accurate to reflect the evolutionary origin of the *Blp* trait.

## Materials and Methods

### Plant Materials and Genomic DNA Extraction

Australian barley variety Hindmarsh (yellow lemma and pericarp) and a wild barley line W1 (black lemma and pericarp) were crossed. A population of 433 DH lines was derived from the F1 using the method described by Hayes et al. (2003). Another three black and yellow segregating F5 populations: TH1 (380 lines), TH2 (313 lines), and TH5 (316 lines) were obtained from the barley germplasm collection at the Department of Agriculture and Food of Western Australia. The phenotype of grain color was assessed visually by eye. Inheritance Chi square analysis was performed using the GraphPad online tool (https://graphpad.com/quickcalcs/chisquared1.cfm). Genomic DNA was extracted from the seeds using the cetyltrimethylammonium bromide (CTAB) method (Doyle, 1987).

### SNP Genotyping and Construction of Preliminary Marker Linkage Map

SNP markers from the Golden Gate barley OPA snp marker panel (Illumina, California, United States) were used to screen for polymorphism between parent lines Hindmarsh and W1. Ninety five polymorphic SNP markers evenly distributed across chromosome 1-7H were used to analyze 188 DH lines (100 black and 88 yellow). The genotype and phenotype data were collected and input into JoinMap 4.0 software (Van Ooijen, 2006) for marker linkage group analyses (LOD = 5). The multipoint maximum likelihood mapping algorithm was used for the calculation of the genetic distance. The SNP marker linkage map was created using the integrated MapChart (Voorrips, 2002) program in JoinMap 4.0 (Van Ooijen, 2006).

### New Indel Markers Design and Fine Mapping of the *Blp* Gene

The designing of new indel markers was facilitated by the new barley genome sequencing project (Mascher et al., 2017). The *Blp* gene was found to be closely linked to SNP marker 1_0722 in the preliminary mapping. In the first round of fine mapping, genetic region around 10 Mb upstream and downstream of marker 1_0722 were searched. Indel markers were designed based on sequence alignment of Morex, Barke and Bowman (obtained from the IPK barley genome database (http://webblast.ipk-gatersleben.de/barley/). These markers were used to analyze the DH population. In the second round of fine mapping, Indel markers were designed between markers MC_48595 and MC_50703. Polymorphism was screened by using the BSA method by pooling the genomic DNA of four black lines and four yellow lines. The TH1, TH2, and TH5 F5 populations were genotyped in search for recombinant lines. Genotyping was performed by standard PCR. The PCR products were run on either 2.5% agarose gel or 6% sodium dodecyl sulfate-polyacrylamide gel (SDS-PAGE).

### Gene Annotation and Candidate Gene Analysis

The information of the candidate genes in the target genetic region were extracted from the new barley genome annotation (Mascher et al., 2017). The amino acid sequence encoded by each candidate gene was searched for close homologs using the Uniprot database (http://www.uniprot.org/). Functional domain analyses were performed using the InterPro online tool (http://www.ebi.ac.uk/interpro/). The reviewed entry hits from Uniprot were used as references for the functional prediction.

### Genotype-Based Clustering Analysis

Indel markers were designed in the genetic region surrounding the target *Blp* gene and were screened for polymorphism between Hindmarsh and W1. These markers were used to analyze 178 barley accessions collected from a diverse geographical locations, spanning the North Africa, the Near East Fertile Crescent, Tibet and East Asia. The genotype for each marker was scored based on their PCR product size and was input into MEGA 7.0 software (Kumar et al., 2016) to generate the distance matrix. The heterozygous genotype was uniformly treated as unknown. An unrooted phylogenetic tree was inferred using MEGA 7.0 (Kumar et al., 2016) with the Neighbor Joining method. Branching support was calculated by the Internal Branching Test method for 1,000 times.

### Genome Re-sequencing Analyses

Four samples including AC_Metcalfe, Baudin, W1, and X1 were re-sequenced using the whole genome short-gun sequencing strategy, with 20–30 times genome coverage. Clean reads of each sample were mapped against the latest barley pseudo-molecular assembly with BWA-MEM program (Li, 2013) arXiv:1303.3997 with default parameters. SNPs and Indels for each sample were detected using the Genome Analysis ToolKit (McKenna et al., 2010), then strict filtration was performed based on the base quality, mapping quality and supporting reads depth. SNPs and indels detected in both black lemma barley W1 and X1 in the target region were analyzed.

## Data Availability

The barley re-sequencing data reported in this study has been deposited in NCBI database under the project code: PRJNA324520.

## Author Contributions

CL and YX conceived the research. YJ wrote the manuscript. ZL designed the primers with the assistance of CT. ZL performed the glasshouse trials and genetic mapping work. YJ carried out the genotype clustering analyses. CT and YJ performed the sequence variation analyses. X-QZ and SW assisted with the molecular mapping. TA, X-QZ, and SB contributed to the seed collection and population development. FD, GZ, and DS contributed to the Tibetan barley germplasm collection and sequencing. CL supervised the project and made critical suggestions on the manuscript development.

### Conflict of Interest Statement

The authors declare that the research was conducted in the absence of any commercial or financial relationships that could be construed as a potential conflict of interest.
