# Supplementary material for: Genetic Mapping and Evolutionary Analyses of the Black Grain Trait in Barley
Source: Front Plant Sci. 2019 Jan 8;9:1921. doi: 10.3389/fpls.2018.01921 (PMC6331406; doi:10.3389/fpls.2018.01921)
Supplement: Supplementary file 1 [file Presentation_1.pdf]

Genetic mapping and evolutionary analyses of the black grain trait in barley

Zhoukai Long[1,2], Yong Jia[2], Cong Tan[2], Xiao-Qi Zhang[2], Tefera Angessa[2], Sue Broughton[3], Sharon Westcott[3], Fei Dai[4], Guoping Zhang[4], Dongfa Sun[5], Yanhao Xu[1], Chengdao Li[2,3]

[1]Hubei Collaborative Innovation Centre for Grain Industry, Yangtze University, Hubei Jingzhou 434025, China.
[2]Western Barley Genetic Alliance, Murdoch University, Western Australia, 6150, Australia.
[3]Department of Agriculture and Food, Government of Western Australia, South Perth WA6155, Australia
[4]College of Agriculture and Biotechnology, Zhejiang University, Hangzhou, China
[5]College of Plant Science, Huazhong Agricultural University, Wuhan, China
* C.Li@murdoch.edu.au

Additional files

Additional file 1. The preliminary linkage map generated from the SNP genotyping results of 188 DH lines. The *Blp* gene was mapped to chromosome 1H.

Additional file 2. Genotyping results of the DH and F5 RIL populations. (Excel format)

Additional file 3. Sequence variations between AC_Metcalfe/Baudin and W1/X in the target genetic region.

Additional file 4. List of barley accessions used for genotype-based clustering analyses and genotype results.

Additional file 5. Representative gel picture of the markers used for genotype clustering analyses.

Additional file 1. The preliminary linkage map generated from the SNP genotyping results of 188 DH lines. The Blp gene was mapped to chromosome 1H.

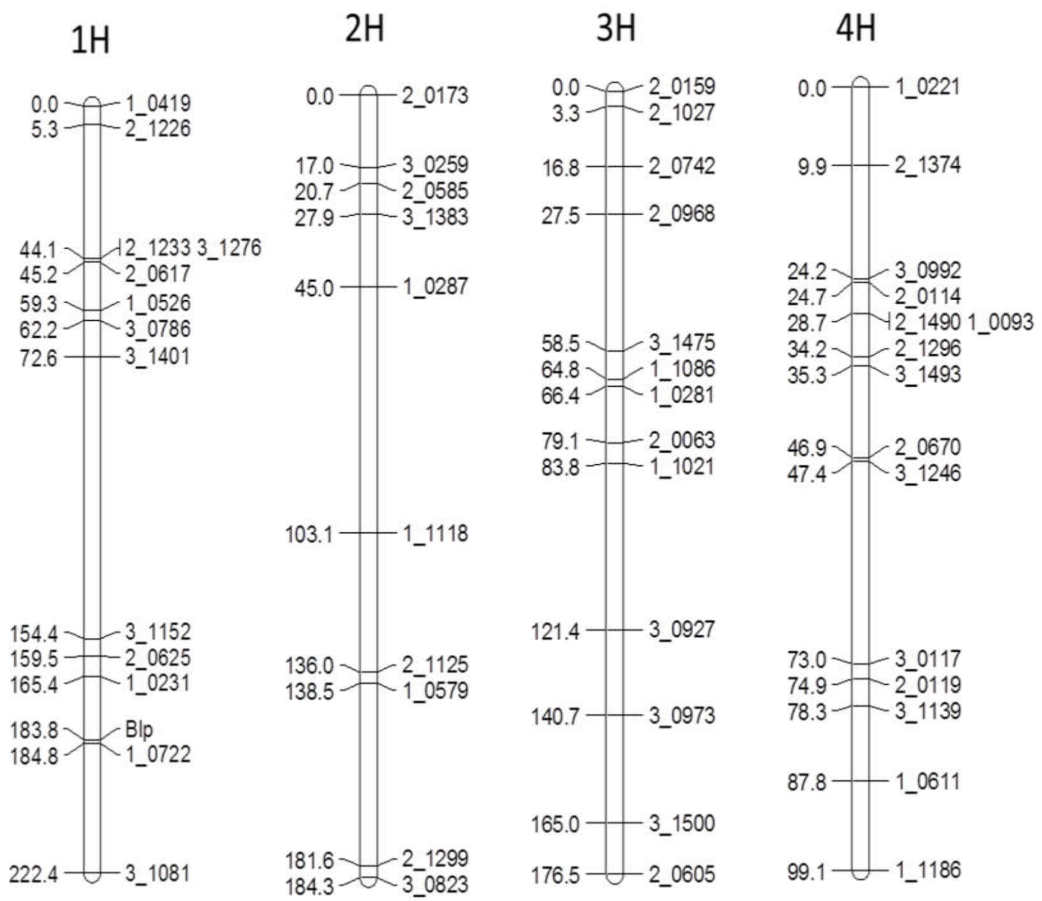

Additional file 2. Genotyping results of the DH and F5 RIL populations. (as separate excel file)

**Additional file 3. Sequence variations between AC_Metcalfe/Baudin and W1/X in the target genetic region.**									
0: represent Morex genotype			1: alternative genotype						
**variant type**									
**Abr**	**Full**								
3PU	3_prime_UTR_variant								
5PUP	5_prime_UTR_premature_start_codon_gain_variant								
5PU	5_prime_UTR_variant								
DII	disruptive_inframe_insertion								
DG	downstream_gene_variant								
FS	frameshift_variant								
IR	intergenic_region								
MS	missense_variant								
SR	splice_region_variant								
SRI	splice_region_variant&intron_variant								
SN	synonymous_variant								
US	upstream_gene_variant								
**CHR**	**POS**	**REF (0)**	**ALT (1)**	**AC_Metacalfe**	**Baudin**	**W1**	**X1**	**Variant Type**	**Gene indication**
chr1H	537907327	C	T	0|0	0|0	1|1	1|1	IR	HORVU1Hr1G086690-HORVU1Hr1G086710
chr1H	537917269	G	A	0|0	0|0	1|1	1|1	IR	HORVU1Hr1G086690-HORVU1Hr1G086710
chr1H	538060250	C	T	0|0	0|0	1|1	1|1	IR	HORVU1Hr1G086750-HORVU1Hr1G086760
chr1H	538077434	G	C	0|0	0|0	1|1	1|1	IR	HORVU1Hr1G086750-HORVU1Hr1G086760
chr1H	538112134	C	G	0|0	0|0	1|1	1|1	DG	HORVU1Hr1G086760
chr1H	538112306	G	C	0|0	0|0	1|1	1|1	DG	HORVU1Hr1G086760
chr1H	538112657	T	TA	0|0	0|0	1|1	1|1	DG	HORVU1Hr1G086760
chr1H	538112843	G	T	0|0	0|0	1|1	1|1	3PU	HORVU1Hr1G086760
chr1H	538114719	T	G	0|0	0|0	1|1	1|1	US	HORVU1Hr1G086760
chr1H	538114736	C	G	0|0	0|0	1|1	1|1	US	HORVU1Hr1G086760
chr1H	538115094	T	A	0|0	0|0	1|1	1|1	US	HORVU1Hr1G086760
chr1H	538115098	A	G	0|0	0|0	1|1	1|1	US	HORVU1Hr1G086760
chr1H	538115982	C	T	0|0	0|0	1|1	1|1	US	HORVU1Hr1G086760
chr1H	538116000	G	A	0|0	0|0	1|1	1|1	US	HORVU1Hr1G086760
chr1H	538116006	T	G	0|0	0|0	1|1	1|1	US	HORVU1Hr1G086760
chr1H	538116333	G	A	0|0	0|0	1|1	1|1	US	HORVU1Hr1G086760
chr1H	538116649	C	T	0|0	0|0	1|1	1|1	US	HORVU1Hr1G086760

chr1H	538116757	T	C	0|0	0|0	1|1	1|1	US	HORVU1Hr1G086760
chr1H	538116828	C	G	0|0	0|0	1|1	1|1	US	HORVU1Hr1G086760
chr1H	538117002	A	G	0|0	0|0	1|1	1|1	US	HORVU1Hr1G086760
chr1H	538117149	T	C	0|0	0|0	1|1	1|1	US	HORVU1Hr1G086760
chr1H	538117214	C	T	0|0	0|0	1|1	1|1	US	HORVU1Hr1G086760
chr1H	538117248	C	A	0|0	0|0	1|1	1|1	US	HORVU1Hr1G086760
chr1H	538117330	A	C	0|0	0|0	1|1	1|1	US	HORVU1Hr1G086760
chr1H	538117527	C	T	0|0	0|0	1|1	1|1	US	HORVU1Hr1G086760
chr1H	538117535	G	A	0|0	0|0	1|1	1|1	US	HORVU1Hr1G086760
chr1H	538117536	G	C	0|0	0|0	1|1	1|1	US	HORVU1Hr1G086760
chr1H	538117597	T	C	0|0	0|0	1|1	1|1	US	HORVU1Hr1G086760
chr1H	538117651	G	A	0|0	0|0	1|1	1|1	US	HORVU1Hr1G086760
chr1H	538117662	G	A	0|0	0|0	1|1	1|1	US	HORVU1Hr1G086760
chr1H	538118106	C	A	0|0	0|0	1|1	1|1	US	HORVU1Hr1G086760
chr1H	538118113	G	C	0|0	0|0	1|1	1|1	US	HORVU1Hr1G086760
chr1H	538118135	G	C	0|0	0|0	1|1	1|1	US	HORVU1Hr1G086760
chr1H	538118248	A	AACTCT	0|0	0|0	1|1	1|1	US	HORVU1Hr1G086760
chr1H	538118294	CACATGA	C	0|0	0|0	1|1	1|1	US	HORVU1Hr1G086760
chr1H	538118302	T	TCG	0|0	0|0	1|1	1|1	US	HORVU1Hr1G086760
chr1H	538118333	C	T	0|0	0|0	1|1	1|1	US	HORVU1Hr1G086760
chr1H	538118409	C	T	0|0	0|0	1|1	1|1	US	HORVU1Hr1G086760
chr1H	538118435	G	C	0|0	0|0	1|1	1|1	US	HORVU1Hr1G086760
chr1H	538118473	G	T	0|0	0|0	1|1	1|1	US	HORVU1Hr1G086760
chr1H	538118493	C	A	0|0	0|0	1|1	1|1	US	HORVU1Hr1G086760
chr1H	538118530	C	A	0|0	0|0	1|1	1|1	US	HORVU1Hr1G086760
chr1H	538118640	T	C	0|0	0|0	1|1	1|1	US	HORVU1Hr1G086760
chr1H	538118664	G	A	0|0	0|0	1|1	1|1	US	HORVU1Hr1G086760
chr1H	538118727	T	A	0|0	0|0	1|1	1|1	US	HORVU1Hr1G086760
chr1H	538119168	T	G	0|0	0|0	1|1	1|1	US	HORVU1Hr1G086760
chr1H	538119298	C	T	0|0	0|0	1|1	1|1	US	HORVU1Hr1G086760
chr1H	538119325	G	A	0|0	0|0	1|1	1|1	US	HORVU1Hr1G086760
chr1H	538119996	G	A	0|0	0|0	1|1	1|1	IR	HORVU1Hr1G086760-HORVU1Hr1G086780
chr1H	538120147	A	G	0|0	0|0	1|1	1|1	IR	HORVU1Hr1G086760-HORVU1Hr1G086780
chr1H	538120293	G	A	0|0	0|0	1|1	1|1	IR	HORVU1Hr1G086760-HORVU1Hr1G086780
chr1H	538120457	CG	C	0|0	0|0	1|1	1|1	IR	HORVU1Hr1G086760-HORVU1Hr1G086780
chr1H	538120899	C	T	0|0	0|0	1|1	1|1	IR	HORVU1Hr1G086760-HORVU1Hr1G086780

chr1H	538120962	AT	A	0|0	0|0	1|1	1|1	IR	HORVU1Hr1G086760-HORVU1Hr1G086780
chr1H	538121807	C	T	0|0	0|0	1|1	1|1	IR	HORVU1Hr1G086760-HORVU1Hr1G086780
chr1H	538121964	G	A	0|0	0|0	1|1	1|1	IR	HORVU1Hr1G086760-HORVU1Hr1G086780
chr1H	538122250	G	A	0|0	0|0	1|1	1|1	IR	HORVU1Hr1G086760-HORVU1Hr1G086780
chr1H	538122374	G	A	0|0	0|0	1|1	1|1	IR	HORVU1Hr1G086760-HORVU1Hr1G086780
chr1H	538122402	C	A	0|0	0|0	1|1	1|1	IR	HORVU1Hr1G086760-HORVU1Hr1G086780
chr1H	538123382	A	C	0|0	0|0	1|1	1|1	IR	HORVU1Hr1G086760-HORVU1Hr1G086780
chr1H	538123840	GTATAT	G	0|0	0|0	1|1	1|1	IR	HORVU1Hr1G086760-HORVU1Hr1G086780
chr1H	538124251	G	A	0|0	0|0	1|1	1|1	IR	HORVU1Hr1G086760-HORVU1Hr1G086780
chr1H	538124262	ATACGG	A	0|0	0|0	1|1	1|1	IR	HORVU1Hr1G086760-HORVU1Hr1G086780
chr1H	538124314	G	C	0|0	0|0	1|1	1|1	IR	HORVU1Hr1G086760-HORVU1Hr1G086780
chr1H	538124443	C	G	0|0	0|0	1|1	1|1	US	HORVU1Hr1G086780
chr1H	538124529	A	G	0|0	0|0	1|1	1|1	US	HORVU1Hr1G086780
chr1H	538125151	C	T	0|0	0|0	1|1	1|1	US	HORVU1Hr1G086780
chr1H	538125156	G	C	0|0	0|0	1|1	1|1	US	HORVU1Hr1G086780
chr1H	538125430	G	A	0|0	0|0	1|1	1|1	US	HORVU1Hr1G086780
chr1H	538125667	TA	T	0|0	0|0	1|1	1|1	US	HORVU1Hr1G086780
chr1H	538125713	A	G	0|0	0|0	1|1	1|1	US	HORVU1Hr1G086780
chr1H	538126376	C	G	0|0	0|0	1|1	1|1	US	HORVU1Hr1G086780
chr1H	538126477	T	C	0|0	0|0	1|1	1|1	US	HORVU1Hr1G086780
chr1H	538126607	A	G	0|0	0|0	1|1	1|1	US	HORVU1Hr1G086780
chr1H	538126917	G	T	0|0	0|0	1|1	1|1	US	HORVU1Hr1G086780
chr1H	538126998	A	C	0|0	0|0	1|1	1|1	US	HORVU1Hr1G086780
chr1H	538127106	G	T	0|0	0|0	1|1	1|1	US	HORVU1Hr1G086780
chr1H	538127435	G	A	0|0	0|0	1|1	1|1	US	HORVU1Hr1G086780
chr1H	538127448	C	T	0|0	0|0	1|1	1|1	US	HORVU1Hr1G086780
chr1H	538127624	G	A	0|0	0|0	1|1	1|1	US	HORVU1Hr1G086780
chr1H	538127784	A	C	0|0	0|0	1|1	1|1	US	HORVU1Hr1G086780
chr1H	538127934	C	A	0|0	0|0	1|1	1|1	US	HORVU1Hr1G086780
chr1H	538129451	G	A	0|0	0|0	1|1	1|1	SN	HORVU1Hr1G086780
chr1H	538129453	G	A	0|0	0|0	1|1	1|1	MS	HORVU1Hr1G086780
chr1H	538129468	C	CAT	0|0	0|0	1|1	1|1	FS	HORVU1Hr1G086780
chr1H	538129479	G	C	0|0	0|0	1|1	1|1	MS	HORVU1Hr1G086780
chr1H	538129687	C	T	0|0	0|0	1|1	1|1	MS	HORVU1Hr1G086780
chr1H	538129789	C	T	0|0	0|0	1|1	1|1	3PU	HORVU1Hr1G086780
chr1H	538129902	T	C	0|0	0|0	1|1	1|1	3PU	HORVU1Hr1G086780

chr1H	538129910	C	A	0|0	0|0	1|1	1|1	3PU	HORVU1Hr1G086780
chr1H	538130187	A	G	0|0	0|0	1|1	1|1	US	HORVU1Hr1G086790
chr1H	538130426	T	A	0|0	0|0	1|1	1|1	US	HORVU1Hr1G086790
chr1H	538130767	C	A	0|0	0|0	1|1	1|1	US	HORVU1Hr1G086790
chr1H	538130821	C	A	0|0	0|0	1|1	1|1	US	HORVU1Hr1G086790
chr1H	538130974	C	T	0|0	0|0	1|1	1|1	US	HORVU1Hr1G086790
chr1H	538131064	G	A	0|0	0|0	1|1	1|1	US	HORVU1Hr1G086790
chr1H	538131176	G	A	0|0	0|0	1|1	1|1	US	HORVU1Hr1G086790
chr1H	538131262	G	A	0|0	0|0	1|1	1|1	US	HORVU1Hr1G086790
chr1H	538131802	T	C	0|0	0|0	1|1	1|1	US	HORVU1Hr1G086790
chr1H	538131847	T	C	0|0	0|0	1|1	1|1	US	HORVU1Hr1G086790
chr1H	538131990	G	A	0|0	0|0	1|1	1|1	US	HORVU1Hr1G086790
chr1H	538132921	C	A	0|0	0|0	1|1	1|1	US	HORVU1Hr1G086790
chr1H	538132987	C	T	0|0	0|0	1|1	1|1	US	HORVU1Hr1G086790
chr1H	538132993	C	T	0|0	0|0	1|1	1|1	US	HORVU1Hr1G086790
chr1H	538133050	C	G	0|0	0|0	1|1	1|1	US	HORVU1Hr1G086790
chr1H	538133079	A	AT	0|0	0|0	1|1	1|1	US	HORVU1Hr1G086790
chr1H	538133091	G	A	0|0	0|0	1|1	1|1	US	HORVU1Hr1G086790
chr1H	538133103	A	T	0|0	0|0	1|1	1|1	US	HORVU1Hr1G086790
chr1H	538133116	G	T	0|0	0|0	1|1	1|1	US	HORVU1Hr1G086790
chr1H	538133191	T	C	0|0	0|0	1|1	1|1	US	HORVU1Hr1G086790
chr1H	538133201	A	G	0|0	0|0	1|1	1|1	US	HORVU1Hr1G086790
chr1H	538133218	C	G	0|0	0|0	1|1	1|1	US	HORVU1Hr1G086790
chr1H	538133228	T	C	0|0	0|0	1|1	1|1	US	HORVU1Hr1G086790
chr1H	538133241	T	TACTA	0|0	0|0	1|1	1|1	US	HORVU1Hr1G086790
chr1H	538133258	A	C	0|0	0|0	1|1	1|1	US	HORVU1Hr1G086790
chr1H	538133972	A	T	0|0	0|0	1|1	1|1	US	HORVU1Hr1G086790
chr1H	538133995	C	CAGT	0|0	0|0	1|1	1|1	US	HORVU1Hr1G086790
chr1H	538134044	C	T	0|0	0|0	1|1	1|1	US	HORVU1Hr1G086790
chr1H	538135203	AGTTTCTTAATTTGAAAGGTGCTACACTG	A	0|0	0|0	1|1	1|1	5PU	HORVU1Hr1G086790
chr1H	538135427	G	T	0|0	0|0	1|1	1|1	MS	HORVU1Hr1G086790
chr1H	538135986	C	T	0|0	0|0	1|1	1|1	DG	HORVU1Hr1G086790
chr1H	538136003	G	C	0|0	0|0	1|1	1|1	DG	HORVU1Hr1G086790
chr1H	538136151	G	T	0|0	0|0	1|1	1|1	DG	HORVU1Hr1G086790
chr1H	538136229	C	T	0|0	0|0	1|1	1|1	DG	HORVU1Hr1G086790
chr1H	538136462	A	T	0|0	0|0	1|1	1|1	DG	HORVU1Hr1G086790

chr1H	538136543	G	A	0|0	0|0	1|1	1|1	DG	HORVU1Hr1G086790
chr1H	538136810	G	A	0|0	0|0	1|1	1|1	DG	HORVU1Hr1G086790
chr1H	538136950	G	C	0|0	0|0	1|1	1|1	DG	HORVU1Hr1G086790
chr1H	538136952	C	T	0|0	0|0	1|1	1|1	DG	HORVU1Hr1G086790
chr1H	538136958	C	T	0|0	0|0	1|1	1|1	DG	HORVU1Hr1G086790
chr1H	538137081	C	T	0|0	0|0	1|1	1|1	DG	HORVU1Hr1G086790
chr1H	538137351	T	A	0|0	0|0	1|1	1|1	DG	HORVU1Hr1G086790
chr1H	538137368	C	T	0|0	0|0	1|1	1|1	DG	HORVU1Hr1G086790
chr1H	538137490	G	A	0|0	0|0	1|1	1|1	DG	HORVU1Hr1G086790
chr1H	538137559	C	T	0|0	0|0	1|1	1|1	DG	HORVU1Hr1G086790
chr1H	538138520	T	C	0|0	0|0	1|1	1|1	DG	HORVU1Hr1G086790
chr1H	538138622	G	A	0|0	0|0	1|1	1|1	DG	HORVU1Hr1G086790
chr1H	538138913	AG	A	0|0	0|0	1|1	1|1	DG	HORVU1Hr1G086790
chr1H	538138950	G	T	0|0	0|0	1|1	1|1	DG	HORVU1Hr1G086790
chr1H	538139012	C	T	0|0	0|0	1|1	1|1	DG	HORVU1Hr1G086790
chr1H	538139293	G	T	0|0	0|0	1|1	1|1	DG	HORVU1Hr1G086790
chr1H	538139414	C	G	0|0	0|0	1|1	1|1	DG	HORVU1Hr1G086790
chr1H	538139479	C	G	0|0	0|0	1|1	1|1	DG	HORVU1Hr1G086790
chr1H	538139986	A	T	0|0	0|0	1|1	1|1	DG	HORVU1Hr1G086790
chr1H	538141737	C	G	0|0	0|0	1|1	1|1	IR	HORVU1Hr1G086790-HORVU1Hr1G086810
chr1H	538141782	G	A	0|0	0|0	1|1	1|1	IR	HORVU1Hr1G086790-HORVU1Hr1G086810
chr1H	538141902	A	G	0|0	0|0	1|1	1|1	IR	HORVU1Hr1G086790-HORVU1Hr1G086810
chr1H	538141954	C	T	0|0	0|0	1|1	1|1	IR	HORVU1Hr1G086790-HORVU1Hr1G086810
chr1H	538141972	G	A	0|0	0|0	1|1	1|1	IR	HORVU1Hr1G086790-HORVU1Hr1G086810
chr1H	538141978	C	T	0|0	0|0	1|1	1|1	IR	HORVU1Hr1G086790-HORVU1Hr1G086810
chr1H	538142001	C	T	0|0	0|0	1|1	1|1	IR	HORVU1Hr1G086790-HORVU1Hr1G086810
chr1H	538142218	T	G	0|0	0|0	1|1	1|1	IR	HORVU1Hr1G086790-HORVU1Hr1G086810
chr1H	538142704	A	G	0|0	0|0	1|1	1|1	IR	HORVU1Hr1G086790-HORVU1Hr1G086810
chr1H	538142714	C	T	0|0	0|0	1|1	1|1	IR	HORVU1Hr1G086790-HORVU1Hr1G086810
chr1H	538142836	C	T	0|0	0|0	1|1	1|1	IR	HORVU1Hr1G086790-HORVU1Hr1G086810
chr1H	538142954	G	A	0|0	0|0	1|1	1|1	IR	HORVU1Hr1G086790-HORVU1Hr1G086810
chr1H	538143001	A	G	0|0	0|0	1|1	1|1	IR	HORVU1Hr1G086790-HORVU1Hr1G086810
chr1H	538143029	C	T	0|0	0|0	1|1	1|1	IR	HORVU1Hr1G086790-HORVU1Hr1G086810
chr1H	538143184	C	T	0|0	0|0	1|1	1|1	IR	HORVU1Hr1G086790-HORVU1Hr1G086810
chr1H	538143276	G	A	0|0	0|0	1|1	1|1	IR	HORVU1Hr1G086790-HORVU1Hr1G086810
chr1H	538143385	C	T	0|0	0|0	1|1	1|1	IR	HORVU1Hr1G086790-HORVU1Hr1G086810

chr1H	538143874	C	A	0|0	0|0	1|1	1|1	IR	HORVU1Hr1G086790-HORVU1Hr1G086810
chr1H	538143909	G	T	0|0	0|0	1|1	1|1	IR	HORVU1Hr1G086790-HORVU1Hr1G086810
chr1H	538143932	C	T	0|0	0|0	1|1	1|1	IR	HORVU1Hr1G086790-HORVU1Hr1G086810
chr1H	538144145	A	T	0|0	0|0	1|1	1|1	IR	HORVU1Hr1G086790-HORVU1Hr1G086810
chr1H	538144361	A	G	0|0	0|0	1|1	1|1	IR	HORVU1Hr1G086790-HORVU1Hr1G086810
chr1H	538144380	C	T	0|0	0|0	1|1	1|1	IR	HORVU1Hr1G086790-HORVU1Hr1G086810
chr1H	538144460	C	T	0|0	0|0	1|1	1|1	IR	HORVU1Hr1G086790-HORVU1Hr1G086810
chr1H	538144479	C	T	0|0	0|0	1|1	1|1	IR	HORVU1Hr1G086790-HORVU1Hr1G086810
chr1H	538144605	G	A	0|0	0|0	1|1	1|1	IR	HORVU1Hr1G086790-HORVU1Hr1G086810
chr1H	538144888	C	T	0|0	0|0	1|1	1|1	IR	HORVU1Hr1G086790-HORVU1Hr1G086810
chr1H	538145346	C	G	0|0	0|0	1|1	1|1	IR	HORVU1Hr1G086790-HORVU1Hr1G086810
chr1H	538145431	T	C	0|0	0|0	1|1	1|1	IR	HORVU1Hr1G086790-HORVU1Hr1G086810
chr1H	538145491	T	C	0|0	0|0	1|1	1|1	IR	HORVU1Hr1G086790-HORVU1Hr1G086810
chr1H	538145785	C	T	0|0	0|0	1|1	1|1	IR	HORVU1Hr1G086790-HORVU1Hr1G086810
chr1H	538145992	A	G	0|0	0|0	1|1	1|1	IR	HORVU1Hr1G086790-HORVU1Hr1G086810
chr1H	538145993	C	A	0|0	0|0	1|1	1|1	IR	HORVU1Hr1G086790-HORVU1Hr1G086810
chr1H	538146060	C	A	0|0	0|0	1|1	1|1	IR	HORVU1Hr1G086790-HORVU1Hr1G086810
chr1H	538146736	G	T	0|0	0|0	1|1	1|1	IR	HORVU1Hr1G086790-HORVU1Hr1G086810
chr1H	538146982	C	T	0|0	0|0	1|1	1|1	IR	HORVU1Hr1G086790-HORVU1Hr1G086810
chr1H	538147230	GC	G	0|0	0|0	1|1	1|1	IR	HORVU1Hr1G086790-HORVU1Hr1G086810
chr1H	538147305	G	A	0|0	0|0	1|1	1|1	IR	HORVU1Hr1G086790-HORVU1Hr1G086810
chr1H	538147363	G	C	0|0	0|0	1|1	1|1	IR	HORVU1Hr1G086790-HORVU1Hr1G086810
chr1H	538147418	T	C	0|0	0|0	1|1	1|1	IR	HORVU1Hr1G086790-HORVU1Hr1G086810
chr1H	538147445	A	C	0|0	0|0	1|1	1|1	IR	HORVU1Hr1G086790-HORVU1Hr1G086810
chr1H	538147891	A	G	0|0	0|0	1|1	1|1	IR	HORVU1Hr1G086790-HORVU1Hr1G086810
chr1H	538148024	T	C	0|0	0|0	1|1	1|1	IR	HORVU1Hr1G086790-HORVU1Hr1G086810
chr1H	538148037	T	C	0|0	0|0	1|1	1|1	IR	HORVU1Hr1G086790-HORVU1Hr1G086810
chr1H	538148196	C	G	0|0	0|0	1|1	1|1	IR	HORVU1Hr1G086790-HORVU1Hr1G086810
chr1H	538148329	A	G	0|0	0|0	1|1	1|1	IR	HORVU1Hr1G086790-HORVU1Hr1G086810
chr1H	538148376	G	A	0|0	0|0	1|1	1|1	IR	HORVU1Hr1G086790-HORVU1Hr1G086810
chr1H	538148391	G	C	0|0	0|0	1|1	1|1	IR	HORVU1Hr1G086790-HORVU1Hr1G086810
chr1H	538148445	A	C	0|0	0|0	1|1	1|1	IR	HORVU1Hr1G086790-HORVU1Hr1G086810
chr1H	538148468	C	T	0|0	0|0	1|1	1|1	IR	HORVU1Hr1G086790-HORVU1Hr1G086810
chr1H	538148472	C	T	0|0	0|0	1|1	1|1	IR	HORVU1Hr1G086790-HORVU1Hr1G086810
chr1H	538148506	G	A	0|0	0|0	1|1	1|1	IR	HORVU1Hr1G086790-HORVU1Hr1G086810
chr1H	538148509	A	G	0|0	0|0	1|1	1|1	IR	HORVU1Hr1G086790-HORVU1Hr1G086810

chr1H	538148515	A	G	0|0	0|0	1|1	1|1	IR	HORVU1Hr1G086790-HORVU1Hr1G086810
chr1H	538148517	C	T	0|0	0|0	1|1	1|1	IR	HORVU1Hr1G086790-HORVU1Hr1G086810
chr1H	538148531	C	T	0|0	0|0	1|1	1|1	IR	HORVU1Hr1G086790-HORVU1Hr1G086810
chr1H	538148539	C	T	0|0	0|0	1|1	1|1	IR	HORVU1Hr1G086790-HORVU1Hr1G086810
chr1H	538148541	A	G	0|0	0|0	1|1	1|1	IR	HORVU1Hr1G086790-HORVU1Hr1G086810
chr1H	538148583	C	A	0|0	0|0	1|1	1|1	IR	HORVU1Hr1G086790-HORVU1Hr1G086810
chr1H	538148584	T	G	0|0	0|0	1|1	1|1	IR	HORVU1Hr1G086790-HORVU1Hr1G086810
chr1H	538148593	G	T	0|0	0|0	1|1	1|1	IR	HORVU1Hr1G086790-HORVU1Hr1G086810
chr1H	538148650	TAC	T	0|0	0|0	1|1	1|1	IR	HORVU1Hr1G086790-HORVU1Hr1G086810
chr1H	538148668	G	C	0|0	0|0	1|1	1|1	IR	HORVU1Hr1G086790-HORVU1Hr1G086810
chr1H	538150924	C	CA	0|0	0|0	1|1	1|1	IR	HORVU1Hr1G086790-HORVU1Hr1G086810
chr1H	538150989	C	T	0|0	0|0	1|1	1|1	IR	HORVU1Hr1G086790-HORVU1Hr1G086810
chr1H	538151346	C	CCTAG	0|0	0|0	1|1	1|1	IR	HORVU1Hr1G086790-HORVU1Hr1G086810
chr1H	538151387	G	GTTTCGTCC	0|0	0|0	1|1	1|1	IR	HORVU1Hr1G086790-HORVU1Hr1G086810
chr1H	538151392	GA	G	0|0	0|0	1|1	1|1	IR	HORVU1Hr1G086790-HORVU1Hr1G086810
chr1H	538151737	A	C	0|0	0|0	1|1	1|1	IR	HORVU1Hr1G086790-HORVU1Hr1G086810
chr1H	538152174	A	T	0|0	0|0	1|1	1|1	IR	HORVU1Hr1G086790-HORVU1Hr1G086810
chr1H	538152303	T	C	0|0	0|0	1|1	1|1	IR	HORVU1Hr1G086790-HORVU1Hr1G086810
chr1H	538152454	T	C	0|0	0|0	1|1	1|1	IR	HORVU1Hr1G086790-HORVU1Hr1G086810
chr1H	538152484	C	T	0|0	0|0	1|1	1|1	IR	HORVU1Hr1G086790-HORVU1Hr1G086810
chr1H	538152492	T	G	0|0	0|0	1|1	1|1	IR	HORVU1Hr1G086790-HORVU1Hr1G086810
chr1H	538152716	T	G	0|0	0|0	1|1	1|1	IR	HORVU1Hr1G086790-HORVU1Hr1G086810
chr1H	538152763	C	T	0|0	0|0	1|1	1|1	IR	HORVU1Hr1G086790-HORVU1Hr1G086810
chr1H	538152782	TCCTCCACCTCGGTGAAG	T	0|0	0|0	1|1	1|1	IR	HORVU1Hr1G086790-HORVU1Hr1G086810
chr1H	538152819	G	A	0|0	0|0	1|1	1|1	IR	HORVU1Hr1G086790-HORVU1Hr1G086810
chr1H	538152969	T	C	0|0	0|0	1|1	1|1	IR	HORVU1Hr1G086790-HORVU1Hr1G086810
chr1H	538153005	T	C	0|0	0|0	1|1	1|1	IR	HORVU1Hr1G086790-HORVU1Hr1G086810
chr1H	538153195	T	C	0|0	0|0	1|1	1|1	IR	HORVU1Hr1G086790-HORVU1Hr1G086810
chr1H	538153203	G	A	0|0	0|0	1|1	1|1	IR	HORVU1Hr1G086790-HORVU1Hr1G086810
chr1H	538153238	C	A	0|0	0|0	1|1	1|1	IR	HORVU1Hr1G086790-HORVU1Hr1G086810
chr1H	538153489	T	C	0|0	0|0	1|1	1|1	IR	HORVU1Hr1G086790-HORVU1Hr1G086810
chr1H	538153619	C	G	0|0	0|0	1|1	1|1	IR	HORVU1Hr1G086790-HORVU1Hr1G086810
chr1H	538154142	G	A	0|0	0|0	1|1	1|1	IR	HORVU1Hr1G086790-HORVU1Hr1G086810
chr1H	538154198	C	T	0|0	0|0	1|1	1|1	IR	HORVU1Hr1G086790-HORVU1Hr1G086810
chr1H	538154314	T	A	0|0	0|0	1|1	1|1	IR	HORVU1Hr1G086790-HORVU1Hr1G086810
chr1H	538154405	A	AGG	0|0	0|0	1|1	1|1	IR	HORVU1Hr1G086790-HORVU1Hr1G086810

chr1H	538154533	C	G	0|0	0|0	1|1	1|1	IR	HORVU1Hr1G086790-HORVU1Hr1G086810
chr1H	538154564	T	C	0|0	0|0	1|1	1|1	IR	HORVU1Hr1G086790-HORVU1Hr1G086810
chr1H	538155001	C	T	0|0	0|0	1|1	1|1	IR	HORVU1Hr1G086790-HORVU1Hr1G086810
chr1H	538155259	G	C	0|0	0|0	1|1	1|1	IR	HORVU1Hr1G086790-HORVU1Hr1G086810
chr1H	538155343	T	C	0|0	0|0	1|1	1|1	IR	HORVU1Hr1G086790-HORVU1Hr1G086810
chr1H	538155524	C	T	0|0	0|0	1|1	1|1	IR	HORVU1Hr1G086790-HORVU1Hr1G086810
chr1H	538155670	G	A	0|0	0|0	1|1	1|1	IR	HORVU1Hr1G086790-HORVU1Hr1G086810
chr1H	538155707	C	T	0|0	0|0	1|1	1|1	IR	HORVU1Hr1G086790-HORVU1Hr1G086810
chr1H	538155852	T	A	0|0	0|0	1|1	1|1	IR	HORVU1Hr1G086790-HORVU1Hr1G086810
chr1H	538155923	G	A	0|0	0|0	1|1	1|1	IR	HORVU1Hr1G086790-HORVU1Hr1G086810
chr1H	538155924	A	T	0|0	0|0	1|1	1|1	IR	HORVU1Hr1G086790-HORVU1Hr1G086810
chr1H	538156117	A	G	0|0	0|0	1|1	1|1	IR	HORVU1Hr1G086790-HORVU1Hr1G086810
chr1H	538156262	A	T	0|0	0|0	1|1	1|1	IR	HORVU1Hr1G086790-HORVU1Hr1G086810
chr1H	538156306	C	T	0|0	0|0	1|1	1|1	IR	HORVU1Hr1G086790-HORVU1Hr1G086810
chr1H	538156382	G	A	0|0	0|0	1|1	1|1	IR	HORVU1Hr1G086790-HORVU1Hr1G086810
chr1H	538156394	A	G	0|0	0|0	1|1	1|1	IR	HORVU1Hr1G086790-HORVU1Hr1G086810
chr1H	538156624	G	A	0|0	0|0	1|1	1|1	IR	HORVU1Hr1G086790-HORVU1Hr1G086810
chr1H	538156656	G	A	0|0	0|0	1|1	1|1	IR	HORVU1Hr1G086790-HORVU1Hr1G086810
chr1H	538156684	G	C	0|0	0|0	1|1	1|1	IR	HORVU1Hr1G086790-HORVU1Hr1G086810
chr1H	538157549	G	A	0|0	0|0	1|1	1|1	IR	HORVU1Hr1G086790-HORVU1Hr1G086810
chr1H	538157947	A	G	0|0	0|0	1|1	1|1	IR	HORVU1Hr1G086790-HORVU1Hr1G086810
chr1H	538157958	G	T	0|0	0|0	1|1	1|1	IR	HORVU1Hr1G086790-HORVU1Hr1G086810
chr1H	538157979	CTCA	C	0|0	0|0	1|1	1|1	IR	HORVU1Hr1G086790-HORVU1Hr1G086810
chr1H	538158023	C	A	0|0	0|0	1|1	1|1	IR	HORVU1Hr1G086790-HORVU1Hr1G086810
chr1H	538158518	A	AT	0|0	0|0	1|1	1|1	IR	HORVU1Hr1G086790-HORVU1Hr1G086810
chr1H	538158713	G	T	0|0	0|0	1|1	1|1	IR	HORVU1Hr1G086790-HORVU1Hr1G086810
chr1H	538158871	G	A	0|0	0|0	1|1	1|1	IR	HORVU1Hr1G086790-HORVU1Hr1G086810
chr1H	538159071	T	G	0|0	0|0	1|1	1|1	IR	HORVU1Hr1G086790-HORVU1Hr1G086810
chr1H	538159122	T	G	0|0	0|0	1|1	1|1	IR	HORVU1Hr1G086790-HORVU1Hr1G086810
chr1H	538159128	T	A	0|0	0|0	1|1	1|1	IR	HORVU1Hr1G086790-HORVU1Hr1G086810
chr1H	538159161	A	T	0|0	0|0	1|1	1|1	IR	HORVU1Hr1G086790-HORVU1Hr1G086810
chr1H	538159977	G	A	0|0	0|0	1|1	1|1	IR	HORVU1Hr1G086790-HORVU1Hr1G086810
chr1H	538166030	T	A	0|0	0|0	1|1	1|1	SN	HORVU1Hr1G086810
chr1H	538166971	C	T	0|0	0|0	1|1	1|1	SN	HORVU1Hr1G086810
chr1H	538177115	C	T	0|0	0|0	1|1	1|1	IR	HORVU1Hr1G086810-HORVU1Hr1G086850
chr1H	538229875	G	C	0|0	0|0	1|1	1|1	IR	HORVU1Hr1G086810-HORVU1Hr1G086850

chr1H	538230035	AG	A	0|0	0|0	1|1	1|1	IR	HORVU1Hr1G086810-HORVU1Hr1G086850
chr1H	538230293	T	C	0|0	0|0	1|1	1|1	IR	HORVU1Hr1G086810-HORVU1Hr1G086850
chr1H	538230596	G	T	0|0	0|0	1|1	1|1	IR	HORVU1Hr1G086810-HORVU1Hr1G086850
chr1H	538230818	G	A	0|0	0|0	1|1	1|1	IR	HORVU1Hr1G086810-HORVU1Hr1G086850
chr1H	538230969	G	C	0|0	0|0	1|1	1|1	IR	HORVU1Hr1G086810-HORVU1Hr1G086850
chr1H	538231198	C	A	0|0	0|0	1|1	1|1	IR	HORVU1Hr1G086810-HORVU1Hr1G086850
chr1H	538231970	G	A	0|0	0|0	1|1	1|1	IR	HORVU1Hr1G086810-HORVU1Hr1G086850
chr1H	538232138	G	A	0|0	0|0	1|1	1|1	IR	HORVU1Hr1G086810-HORVU1Hr1G086850
chr1H	538232220	G	A	0|0	0|0	1|1	1|1	IR	HORVU1Hr1G086810-HORVU1Hr1G086850
chr1H	538232467	T	G	0|0	0|0	1|1	1|1	IR	HORVU1Hr1G086810-HORVU1Hr1G086850
chr1H	538232546	G	A	0|0	0|0	1|1	1|1	IR	HORVU1Hr1G086810-HORVU1Hr1G086850
chr1H	538232844	C	T	0|0	0|0	1|1	1|1	IR	HORVU1Hr1G086810-HORVU1Hr1G086850
chr1H	538232956	G	A	0|0	0|0	1|1	1|1	IR	HORVU1Hr1G086810-HORVU1Hr1G086850
chr1H	538233096	C	T	0|0	0|0	1|1	1|1	IR	HORVU1Hr1G086810-HORVU1Hr1G086850
chr1H	538233378	G	T	0|0	0|0	1|1	1|1	IR	HORVU1Hr1G086810-HORVU1Hr1G086850
chr1H	538233532	C	T	0|0	0|0	1|1	1|1	IR	HORVU1Hr1G086810-HORVU1Hr1G086850
chr1H	538233641	G	A	0|0	0|0	1|1	1|1	IR	HORVU1Hr1G086810-HORVU1Hr1G086850
chr1H	538233695	C	T	0|0	0|0	1|1	1|1	IR	HORVU1Hr1G086810-HORVU1Hr1G086850
chr1H	538233757	C	A	0|0	0|0	1|1	1|1	IR	HORVU1Hr1G086810-HORVU1Hr1G086850
chr1H	538234067	G	T	0|0	0|0	1|1	1|1	IR	HORVU1Hr1G086810-HORVU1Hr1G086850
chr1H	538234112	C	T	0|0	0|0	1|1	1|1	IR	HORVU1Hr1G086810-HORVU1Hr1G086850
chr1H	538234145	G	A	0|0	0|0	1|1	1|1	IR	HORVU1Hr1G086810-HORVU1Hr1G086850
chr1H	538234482	C	G	0|0	0|0	1|1	1|1	IR	HORVU1Hr1G086810-HORVU1Hr1G086850
chr1H	538234550	A	T	0|0	0|0	1|1	1|1	IR	HORVU1Hr1G086810-HORVU1Hr1G086850
chr1H	538234585	C	T	0|0	0|0	1|1	1|1	IR	HORVU1Hr1G086810-HORVU1Hr1G086850
chr1H	538234761	G	A	0|0	0|0	1|1	1|1	IR	HORVU1Hr1G086810-HORVU1Hr1G086850
chr1H	538234955	C	T	0|0	0|0	1|1	1|1	IR	HORVU1Hr1G086810-HORVU1Hr1G086850
chr1H	538235388	A	G	0|0	0|0	1|1	1|1	IR	HORVU1Hr1G086810-HORVU1Hr1G086850
chr1H	538235415	C	T	0|0	0|0	1|1	1|1	IR	HORVU1Hr1G086810-HORVU1Hr1G086850
chr1H	538235417	G	A	0|0	0|0	1|1	1|1	IR	HORVU1Hr1G086810-HORVU1Hr1G086850
chr1H	538235490	C	T	0|0	0|0	1|1	1|1	IR	HORVU1Hr1G086810-HORVU1Hr1G086850
chr1H	538235663	C	T	0|0	0|0	1|1	1|1	IR	HORVU1Hr1G086810-HORVU1Hr1G086850
chr1H	538236171	G	C	0|0	0|0	1|1	1|1	IR	HORVU1Hr1G086810-HORVU1Hr1G086850
chr1H	538236179	G	T	0|0	0|0	1|1	1|1	IR	HORVU1Hr1G086810-HORVU1Hr1G086850
chr1H	538236261	T	A	0|0	0|0	1|1	1|1	IR	HORVU1Hr1G086810-HORVU1Hr1G086850
chr1H	538236262	T	A	0|0	0|0	1|1	1|1	IR	HORVU1Hr1G086810-HORVU1Hr1G086850

chr1H	538236263	T	A	0|0	0|0	1|1	1|1	IR	HORVU1Hr1G086810-HORVU1Hr1G086850
chr1H	538236265	T	A	0|0	0|0	1|1	1|1	IR	HORVU1Hr1G086810-HORVU1Hr1G086850
chr1H	538236285	C	T	0|0	0|0	1|1	1|1	IR	HORVU1Hr1G086810-HORVU1Hr1G086850
chr1H	538236399	C	A	0|0	0|0	1|1	1|1	IR	HORVU1Hr1G086810-HORVU1Hr1G086850
chr1H	538236520	G	T	0|0	0|0	1|1	1|1	IR	HORVU1Hr1G086810-HORVU1Hr1G086850
chr1H	538236809	A	G	0|0	0|0	1|1	1|1	IR	HORVU1Hr1G086810-HORVU1Hr1G086850
chr1H	538236966	G	A	0|0	0|0	1|1	1|1	IR	HORVU1Hr1G086810-HORVU1Hr1G086850
chr1H	538237120	A	G	0|0	0|0	1|1	1|1	IR	HORVU1Hr1G086810-HORVU1Hr1G086850
chr1H	538237308	A	T	0|0	0|0	1|1	1|1	IR	HORVU1Hr1G086810-HORVU1Hr1G086850
chr1H	538237517	G	T	0|0	0|0	1|1	1|1	IR	HORVU1Hr1G086810-HORVU1Hr1G086850
chr1H	538237531	C	T	0|0	0|0	1|1	1|1	IR	HORVU1Hr1G086810-HORVU1Hr1G086850
chr1H	538237635	C	G	0|0	0|0	1|1	1|1	IR	HORVU1Hr1G086810-HORVU1Hr1G086850
chr1H	538237875	G	C	0|0	0|0	1|1	1|1	IR	HORVU1Hr1G086810-HORVU1Hr1G086850
chr1H	538238109	G	T	0|0	0|0	1|1	1|1	IR	HORVU1Hr1G086810-HORVU1Hr1G086850
chr1H	538238206	G	A	0|0	0|0	1|1	1|1	IR	HORVU1Hr1G086810-HORVU1Hr1G086850
chr1H	538238323	G	A	0|0	0|0	1|1	1|1	IR	HORVU1Hr1G086810-HORVU1Hr1G086850
chr1H	538238646	G	A	0|0	0|0	1|1	1|1	IR	HORVU1Hr1G086810-HORVU1Hr1G086850
chr1H	538238827	T	G	0|0	0|0	1|1	1|1	IR	HORVU1Hr1G086810-HORVU1Hr1G086850
chr1H	538238945	G	A	0|0	0|0	1|1	1|1	IR	HORVU1Hr1G086810-HORVU1Hr1G086850
chr1H	538239007	A	G	0|0	0|0	1|1	1|1	IR	HORVU1Hr1G086810-HORVU1Hr1G086850
chr1H	538239045	T	A	0|0	0|0	1|1	1|1	IR	HORVU1Hr1G086810-HORVU1Hr1G086850
chr1H	538239204	C	T	0|0	0|0	1|1	1|1	IR	HORVU1Hr1G086810-HORVU1Hr1G086850
chr1H	538239350	G	A	0|0	0|0	1|1	1|1	IR	HORVU1Hr1G086810-HORVU1Hr1G086850
chr1H	538239408	G	A	0|0	0|0	1|1	1|1	IR	HORVU1Hr1G086810-HORVU1Hr1G086850
chr1H	538239927	T	C	0|0	0|0	1|1	1|1	IR	HORVU1Hr1G086810-HORVU1Hr1G086850
chr1H	538239934	T	C	0|0	0|0	1|1	1|1	IR	HORVU1Hr1G086810-HORVU1Hr1G086850
chr1H	538240436	C	T	0|0	0|0	1|1	1|1	IR	HORVU1Hr1G086810-HORVU1Hr1G086850
chr1H	538240683	C	A	0|0	0|0	1|1	1|1	IR	HORVU1Hr1G086810-HORVU1Hr1G086850
chr1H	538240684	T	G	0|0	0|0	1|1	1|1	IR	HORVU1Hr1G086810-HORVU1Hr1G086850
chr1H	538240706	G	A	0|0	0|0	1|1	1|1	IR	HORVU1Hr1G086810-HORVU1Hr1G086850
chr1H	538240741	C	T	0|0	0|0	1|1	1|1	IR	HORVU1Hr1G086810-HORVU1Hr1G086850
chr1H	538240751	C	T	0|0	0|0	1|1	1|1	IR	HORVU1Hr1G086810-HORVU1Hr1G086850
chr1H	538241099	TG	T	0|0	0|0	1|1	1|1	IR	HORVU1Hr1G086810-HORVU1Hr1G086850
chr1H	538241154	A	C	0|0	0|0	1|1	1|1	IR	HORVU1Hr1G086810-HORVU1Hr1G086850
chr1H	538241406	C	T	0|0	0|0	1|1	1|1	IR	HORVU1Hr1G086810-HORVU1Hr1G086850
chr1H	538241718	G	A	0|0	0|0	1|1	1|1	IR	HORVU1Hr1G086810-HORVU1Hr1G086850

chr1H	538242010	A	G	0|0	0|0	1|1	1|1	IR	HORVU1Hr1G086810-HORVU1Hr1G086850
chr1H	538242389	A	C	0|0	0|0	1|1	1|1	IR	HORVU1Hr1G086810-HORVU1Hr1G086850
chr1H	538242433	G	C	0|0	0|0	1|1	1|1	IR	HORVU1Hr1G086810-HORVU1Hr1G086850
chr1H	538242510	C	G	0|0	0|0	1|1	1|1	IR	HORVU1Hr1G086810-HORVU1Hr1G086850
chr1H	538242622	GC	G	0|0	0|0	1|1	1|1	IR	HORVU1Hr1G086810-HORVU1Hr1G086850
chr1H	538242885	G	T	0|0	0|0	1|1	1|1	IR	HORVU1Hr1G086810-HORVU1Hr1G086850
chr1H	538243168	C	T	0|0	0|0	1|1	1|1	IR	HORVU1Hr1G086810-HORVU1Hr1G086850
chr1H	538243201	G	A	0|0	0|0	1|1	1|1	IR	HORVU1Hr1G086810-HORVU1Hr1G086850
chr1H	538243258	C	A	0|0	0|0	1|1	1|1	IR	HORVU1Hr1G086810-HORVU1Hr1G086850
chr1H	538243721	T	A	0|0	0|0	1|1	1|1	IR	HORVU1Hr1G086810-HORVU1Hr1G086850
chr1H	538243770	C	T	0|0	0|0	1|1	1|1	IR	HORVU1Hr1G086810-HORVU1Hr1G086850
chr1H	538244375	T	C	0|0	0|0	1|1	1|1	IR	HORVU1Hr1G086810-HORVU1Hr1G086850
chr1H	538282820	G	C	0|0	0|0	1|1	1|1	IR	HORVU1Hr1G086810-HORVU1Hr1G086850
chr1H	538282849	C	T	0|0	0|0	1|1	1|1	IR	HORVU1Hr1G086810-HORVU1Hr1G086850
chr1H	538282887	G	A	0|0	0|0	1|1	1|1	IR	HORVU1Hr1G086810-HORVU1Hr1G086850
chr1H	538294083	A	G	0|0	0|0	1|1	1|1	IR	HORVU1Hr1G086810-HORVU1Hr1G086850
chr1H	538294146	C	A	0|0	0|0	1|1	1|1	IR	HORVU1Hr1G086810-HORVU1Hr1G086850
chr1H	538308657	C	T	0|0	0|0	1|1	1|1	IR	HORVU1Hr1G086810-HORVU1Hr1G086850
chr1H	538308673	C	G	0|0	0|0	1|1	1|1	IR	HORVU1Hr1G086810-HORVU1Hr1G086850
chr1H	538308677	G	T	0|0	0|0	1|1	1|1	IR	HORVU1Hr1G086810-HORVU1Hr1G086850
chr1H	538308680	G	C	0|0	0|0	1|1	1|1	IR	HORVU1Hr1G086810-HORVU1Hr1G086850
chr1H	538308738	T	C	0|0	0|0	1|1	1|1	IR	HORVU1Hr1G086810-HORVU1Hr1G086850
chr1H	538308863	A	AG	0|0	0|0	1|1	1|1	IR	HORVU1Hr1G086810-HORVU1Hr1G086850
chr1H	538308996	G	GCTAGTGCA	0|0	0|0	1|1	1|1	IR	HORVU1Hr1G086810-HORVU1Hr1G086850
chr1H	538309039	A	G	0|0	0|0	1|1	1|1	IR	HORVU1Hr1G086810-HORVU1Hr1G086850
chr1H	538309505	C	G	0|0	0|0	1|1	1|1	IR	HORVU1Hr1G086810-HORVU1Hr1G086850
chr1H	538309872	A	G	0|0	0|0	1|1	1|1	IR	HORVU1Hr1G086810-HORVU1Hr1G086850
chr1H	538309905	C	G	0|0	0|0	1|1	1|1	IR	HORVU1Hr1G086810-HORVU1Hr1G086850
chr1H	538310011	G	A	0|0	0|0	1|1	1|1	IR	HORVU1Hr1G086810-HORVU1Hr1G086850
chr1H	538310347	A	G	0|0	0|0	1|1	1|1	IR	HORVU1Hr1G086810-HORVU1Hr1G086850
chr1H	538310541	T	C	0|0	0|0	1|1	1|1	IR	HORVU1Hr1G086810-HORVU1Hr1G086850
chr1H	538310747	ACC	A	0|0	0|0	1|1	1|1	IR	HORVU1Hr1G086810-HORVU1Hr1G086850
chr1H	538313133	G	A	0|0	0|0	1|1	1|1	IR	HORVU1Hr1G086810-HORVU1Hr1G086850
chr1H	538313157	C	T	0|0	0|0	1|1	1|1	IR	HORVU1Hr1G086810-HORVU1Hr1G086850
chr1H	538315883	T	C	0|0	0|0	1|1	1|1	IR	HORVU1Hr1G086810-HORVU1Hr1G086850
chr1H	538316244	G	C	0|0	0|0	1|1	1|1	IR	HORVU1Hr1G086810-HORVU1Hr1G086850

chr1H	538316515	C	T	0|0	0|0	1|1	1|1	IR	HORVU1Hr1G086810-HORVU1Hr1G086850
chr1H	538316604	G	A	0|0	0|0	1|1	1|1	IR	HORVU1Hr1G086810-HORVU1Hr1G086850
chr1H	538316749	T	C	0|0	0|0	1|1	1|1	IR	HORVU1Hr1G086810-HORVU1Hr1G086850
chr1H	538316848	G	A	0|0	0|0	1|1	1|1	IR	HORVU1Hr1G086810-HORVU1Hr1G086850
chr1H	538316873	C	T	0|0	0|0	1|1	1|1	IR	HORVU1Hr1G086810-HORVU1Hr1G086850
chr1H	538317500	C	T	0|0	0|0	1|1	1|1	IR	HORVU1Hr1G086810-HORVU1Hr1G086850
chr1H	538318570	CT	C	0|0	0|0	1|1	1|1	IR	HORVU1Hr1G086810-HORVU1Hr1G086850
chr1H	538318620	C	A	0|0	0|0	1|1	1|1	IR	HORVU1Hr1G086810-HORVU1Hr1G086850
chr1H	538318628	T	C	0|0	0|0	1|1	1|1	IR	HORVU1Hr1G086810-HORVU1Hr1G086850
chr1H	538318703	C	T	0|0	0|0	1|1	1|1	IR	HORVU1Hr1G086810-HORVU1Hr1G086850
chr1H	538318705	G	A	0|0	0|0	1|1	1|1	IR	HORVU1Hr1G086810-HORVU1Hr1G086850
chr1H	538318827	T	C	0|0	0|0	1|1	1|1	IR	HORVU1Hr1G086810-HORVU1Hr1G086850
chr1H	538318919	G	T	0|0	0|0	1|1	1|1	IR	HORVU1Hr1G086810-HORVU1Hr1G086850
chr1H	538319054	C	G	0|0	0|0	1|1	1|1	IR	HORVU1Hr1G086810-HORVU1Hr1G086850
chr1H	538319105	C	T	0|0	0|0	1|1	1|1	IR	HORVU1Hr1G086810-HORVU1Hr1G086850
chr1H	538319558	G	T	0|0	0|0	1|1	1|1	IR	HORVU1Hr1G086810-HORVU1Hr1G086850
chr1H	538319593	T	C	0|0	0|0	1|1	1|1	IR	HORVU1Hr1G086810-HORVU1Hr1G086850
chr1H	538319603	C	T	0|0	0|0	1|1	1|1	IR	HORVU1Hr1G086810-HORVU1Hr1G086850
chr1H	538319758	G	A	0|0	0|0	1|1	1|1	IR	HORVU1Hr1G086810-HORVU1Hr1G086850
chr1H	538319965	A	G	0|0	0|0	1|1	1|1	IR	HORVU1Hr1G086810-HORVU1Hr1G086850
chr1H	538320018	G	A	0|0	0|0	1|1	1|1	IR	HORVU1Hr1G086810-HORVU1Hr1G086850
chr1H	538320258	G	T	0|0	0|0	1|1	1|1	IR	HORVU1Hr1G086810-HORVU1Hr1G086850
chr1H	538320596	G	T	0|0	0|0	1|1	1|1	IR	HORVU1Hr1G086810-HORVU1Hr1G086850
chr1H	538320781	C	A	0|0	0|0	1|1	1|1	IR	HORVU1Hr1G086810-HORVU1Hr1G086850
chr1H	538320937	G	T	0|0	0|0	1|1	1|1	IR	HORVU1Hr1G086810-HORVU1Hr1G086850
chr1H	538321146	C	T	0|0	0|0	1|1	1|1	IR	HORVU1Hr1G086810-HORVU1Hr1G086850
chr1H	538321192	T	C	0|0	0|0	1|1	1|1	IR	HORVU1Hr1G086810-HORVU1Hr1G086850
chr1H	538321232	G	A	0|0	0|0	1|1	1|1	IR	HORVU1Hr1G086810-HORVU1Hr1G086850
chr1H	538321263	G	A	0|0	0|0	1|1	1|1	IR	HORVU1Hr1G086810-HORVU1Hr1G086850
chr1H	538321278	T	C	0|0	0|0	1|1	1|1	IR	HORVU1Hr1G086810-HORVU1Hr1G086850
chr1H	538321321	A	C	0|0	0|0	1|1	1|1	IR	HORVU1Hr1G086810-HORVU1Hr1G086850
chr1H	538321361	A	T	0|0	0|0	1|1	1|1	IR	HORVU1Hr1G086810-HORVU1Hr1G086850
chr1H	538321544	T	C	0|0	0|0	1|1	1|1	IR	HORVU1Hr1G086810-HORVU1Hr1G086850
chr1H	538321919	C	T	0|0	0|0	1|1	1|1	IR	HORVU1Hr1G086810-HORVU1Hr1G086850
chr1H	538322014	C	G	0|0	0|0	1|1	1|1	IR	HORVU1Hr1G086810-HORVU1Hr1G086850
chr1H	538322217	C	G	0|0	0|0	1|1	1|1	IR	HORVU1Hr1G086810-HORVU1Hr1G086850

chr1H	538322398	C	T	0|0	0|0	1|1	1|1	IR	HORVU1Hr1G086810-HORVU1Hr1G086850
chr1H	538322594	G	A	0|0	0|0	1|1	1|1	IR	HORVU1Hr1G086810-HORVU1Hr1G086850
chr1H	538322837	G	A	0|0	0|0	1|1	1|1	IR	HORVU1Hr1G086810-HORVU1Hr1G086850
chr1H	538323001	C	T	0|0	0|0	1|1	1|1	IR	HORVU1Hr1G086810-HORVU1Hr1G086850
chr1H	538324024	G	A	0|0	0|0	1|1	1|1	IR	HORVU1Hr1G086810-HORVU1Hr1G086850
chr1H	538324068	G	A	0|0	0|0	1|1	1|1	IR	HORVU1Hr1G086810-HORVU1Hr1G086850
chr1H	538324460	C	T	0|0	0|0	1|1	1|1	IR	HORVU1Hr1G086810-HORVU1Hr1G086850
chr1H	538324993	T	C	0|0	0|0	1|1	1|1	IR	HORVU1Hr1G086810-HORVU1Hr1G086850
chr1H	538325152	G	C	0|0	0|0	1|1	1|1	IR	HORVU1Hr1G086810-HORVU1Hr1G086850
chr1H	538325318	A	C	0|0	0|0	1|1	1|1	IR	HORVU1Hr1G086810-HORVU1Hr1G086850
chr1H	538325339	A	T	0|0	0|0	1|1	1|1	IR	HORVU1Hr1G086810-HORVU1Hr1G086850
chr1H	538325453	T	A	0|0	0|0	1|1	1|1	IR	HORVU1Hr1G086810-HORVU1Hr1G086850
chr1H	538325686	C	T	0|0	0|0	1|1	1|1	IR	HORVU1Hr1G086810-HORVU1Hr1G086850
chr1H	538326094	C	G	0|0	0|0	1|1	1|1	IR	HORVU1Hr1G086810-HORVU1Hr1G086850
chr1H	538326203	A	C	0|0	0|0	1|1	1|1	IR	HORVU1Hr1G086810-HORVU1Hr1G086850
chr1H	538326328	G	A	0|0	0|0	1|1	1|1	IR	HORVU1Hr1G086810-HORVU1Hr1G086850
chr1H	538326379	G	A	0|0	0|0	1|1	1|1	IR	HORVU1Hr1G086810-HORVU1Hr1G086850
chr1H	538326403	C	A	0|0	0|0	1|1	1|1	IR	HORVU1Hr1G086810-HORVU1Hr1G086850
chr1H	538326564	C	T	0|0	0|0	1|1	1|1	IR	HORVU1Hr1G086810-HORVU1Hr1G086850
chr1H	538326716	T	C	0|0	0|0	1|1	1|1	IR	HORVU1Hr1G086810-HORVU1Hr1G086850
chr1H	538326768	G	T	0|0	0|0	1|1	1|1	IR	HORVU1Hr1G086810-HORVU1Hr1G086850
chr1H	538370084	A	G	0|0	0|0	1|1	1|1	IR	HORVU1Hr1G086850-HORVU1Hr1G086870
chr1H	538371436	G	A	0|0	0|0	1|1	1|1	IR	HORVU1Hr1G086850-HORVU1Hr1G086870
chr1H	538371666	C	T	0|0	0|0	1|1	1|1	IR	HORVU1Hr1G086850-HORVU1Hr1G086870
chr1H	538371673	G	A	0|0	0|0	1|1	1|1	IR	HORVU1Hr1G086850-HORVU1Hr1G086870
chr1H	538371810	T	G	0|0	0|0	1|1	1|1	US	HORVU1Hr1G086870
chr1H	538373372	C	A	0|0	0|0	1|1	1|1	US	HORVU1Hr1G086870
chr1H	538373739	C	A	0|0	0|0	1|1	1|1	US	HORVU1Hr1G086870
chr1H	538374366	A	G	0|0	0|0	1|1	1|1	US	HORVU1Hr1G086870
chr1H	538374547	A	G	0|0	0|0	1|1	1|1	US	HORVU1Hr1G086870
chr1H	538375171	G	A	0|0	0|0	1|1	1|1	US	HORVU1Hr1G086870
chr1H	538377138	T	A	0|0	0|0	1|1	1|1	5PU	HORVU1Hr1G086870
chr1H	538377433	A	T	0|0	0|0	1|1	1|1	US	HORVU1Hr1G086870
chr1H	538379015	C	T	0|0	0|0	1|1	1|1	DG	HORVU1Hr1G086870
chr1H	538379215	T	A	0|0	0|0	1|1	1|1	DG	HORVU1Hr1G086870
chr1H	538379217	T	A	0|0	0|0	1|1	1|1	DG	HORVU1Hr1G086870

chr1H	538379227	C	T	0|0	0|0	1|1	1|1	DG	HORVU1Hr1G086870
chr1H	538381166	T	C	0|0	0|0	1|1	1|1	DG	HORVU1Hr1G086870
chr1H	538382077	AT	A	0|0	0|0	1|1	1|1	3PU	HORVU1Hr1G086890
chr1H	538382580	C	A	0|0	0|0	1|1	1|1	3PU	HORVU1Hr1G086890
chr1H	538382729	G	T	0|0	0|0	1|1	1|1	MS	HORVU1Hr1G086890
chr1H	538382835	A	G	0|0	0|0	1|1	1|1	3PU	HORVU1Hr1G086890
chr1H	538383329	C	T	0|0	0|0	1|1	1|1	SN	HORVU1Hr1G086890
chr1H	538383709	T	C	0|0	0|0	1|1	1|1	US	HORVU1Hr1G086890
chr1H	538384127	G	T	0|0	0|0	1|1	1|1	US	HORVU1Hr1G086890
chr1H	538384228	G	T	0|0	0|0	1|1	1|1	US	HORVU1Hr1G086890
chr1H	538384467	G	A	0|0	0|0	1|1	1|1	MS	HORVU1Hr1G086890
chr1H	538384751	C	T	0|0	0|0	1|1	1|1	MS	HORVU1Hr1G086890
chr1H	538385293	C	T	0|0	0|0	1|1	1|1	MS	HORVU1Hr1G086890
chr1H	538385391	G	A	0|0	0|0	1|1	1|1	SN	HORVU1Hr1G086890
chr1H	538385859	A	C	0|0	0|0	1|1	1|1	US	HORVU1Hr1G086890
chr1H	538386094	A	G	0|0	0|0	1|1	1|1	US	HORVU1Hr1G086890
chr1H	538386787	G	C	0|0	0|0	1|1	1|1	US	HORVU1Hr1G086890
chr1H	538387166	G	A	0|0	0|0	1|1	1|1	US	HORVU1Hr1G086890
chr1H	538387171	T	G	0|0	0|0	1|1	1|1	US	HORVU1Hr1G086890
chr1H	538387486	T	C	0|0	0|0	1|1	1|1	US	HORVU1Hr1G086890
chr1H	538387934	A	T	0|0	0|0	1|1	1|1	US	HORVU1Hr1G086890
chr1H	538388087	A	G	0|0	0|0	1|1	1|1	US	HORVU1Hr1G086890
chr1H	538388447	A	C	0|0	0|0	1|1	1|1	US	HORVU1Hr1G086890
chr1H	538388476	G	A	0|0	0|0	1|1	1|1	US	HORVU1Hr1G086890
chr1H	538390965	T	C	0|0	0|0	1|1	1|1	MS	HORVU1Hr1G086900
chr1H	538391601	G	C	0|0	0|0	1|1	1|1	US	HORVU1Hr1G086890
chr1H	538393857	T	C	0|0	0|0	1|1	1|1	US	HORVU1Hr1G086900
chr1H	538394477	G	A	0|0	0|0	1|1	1|1	US	HORVU1Hr1G086900
chr1H	538394714	C	T	0|0	0|0	1|1	1|1	US	HORVU1Hr1G086900
chr1H	538396557	C	A	0|0	0|0	1|1	1|1	IR	HORVU1Hr1G086900-HORVU1Hr1G086910
chr1H	538396562	G	A	0|0	0|0	1|1	1|1	IR	HORVU1Hr1G086900-HORVU1Hr1G086910
chr1H	538397699	A	G	0|0	0|0	1|1	1|1	IR	HORVU1Hr1G086900-HORVU1Hr1G086910
chr1H	538397996	C	T	0|0	0|0	1|1	1|1	IR	HORVU1Hr1G086900-HORVU1Hr1G086910
chr1H	538398593	C	T	0|0	0|0	1|1	1|1	IR	HORVU1Hr1G086900-HORVU1Hr1G086910
chr1H	538398655	T	G	0|0	0|0	1|1	1|1	IR	HORVU1Hr1G086900-HORVU1Hr1G086910
chr1H	538398765	G	C	0|0	0|0	1|1	1|1	IR	HORVU1Hr1G086900-HORVU1Hr1G086910

chr1H	538399084	C	T	0|0	0|0	1|1	1|1	IR	HORVU1Hr1G086900-HORVU1Hr1G086910
chr1H	538399899	G	A	0|0	0|0	1|1	1|1	IR	HORVU1Hr1G086900-HORVU1Hr1G086910
chr1H	538400034	G	A	0|0	0|0	1|1	1|1	IR	HORVU1Hr1G086900-HORVU1Hr1G086910
chr1H	538400138	G	T	0|0	0|0	1|1	1|1	IR	HORVU1Hr1G086900-HORVU1Hr1G086910
chr1H	538400276	G	A	0|0	0|0	1|1	1|1	IR	HORVU1Hr1G086900-HORVU1Hr1G086910
chr1H	538400408	C	A	0|0	0|0	1|1	1|1	IR	HORVU1Hr1G086900-HORVU1Hr1G086910
chr1H	538400450	G	A	0|0	0|0	1|1	1|1	IR	HORVU1Hr1G086900-HORVU1Hr1G086910
chr1H	538400543	G	A	0|0	0|0	1|1	1|1	IR	HORVU1Hr1G086900-HORVU1Hr1G086910
chr1H	538401741	G	A	0|0	0|0	1|1	1|1	IR	HORVU1Hr1G086900-HORVU1Hr1G086910
chr1H	538402750	G	A	0|0	0|0	1|1	1|1	IR	HORVU1Hr1G086900-HORVU1Hr1G086910
chr1H	538404580	G	C	0|0	0|0	1|1	1|1	IR	HORVU1Hr1G086900-HORVU1Hr1G086910
chr1H	538404667	A	AT	0|0	0|0	1|1	1|1	IR	HORVU1Hr1G086900-HORVU1Hr1G086910
chr1H	538404899	A	T	0|0	0|0	1|1	1|1	IR	HORVU1Hr1G086900-HORVU1Hr1G086910
chr1H	538405071	A	G	0|0	0|0	1|1	1|1	US	HORVU1Hr1G086910
chr1H	538407007	T	C	0|0	0|0	1|1	1|1	US	HORVU1Hr1G086910
chr1H	538407104	G	C	0|0	0|0	1|1	1|1	US	HORVU1Hr1G086910
chr1H	538407394	G	A	0|0	0|0	1|1	1|1	US	HORVU1Hr1G086910
chr1H	538407722	T	C	0|0	0|0	1|1	1|1	US	HORVU1Hr1G086910
chr1H	538407755	G	A	0|0	0|0	1|1	1|1	US	HORVU1Hr1G086910
chr1H	538407998	G	A	0|0	0|0	1|1	1|1	US	HORVU1Hr1G086910
chr1H	538408644	C	T	0|0	0|0	1|1	1|1	US	HORVU1Hr1G086910
chr1H	538409595	G	GA	0|0	0|0	1|1	1|1	US	HORVU1Hr1G086910
chr1H	538409851	A	AG	0|0	0|0	1|1	1|1	US	HORVU1Hr1G086910
chr1H	538410255	G	A	0|0	0|0	1|1	1|1	5PU	HORVU1Hr1G086910
chr1H	538410478	G	T	0|0	0|0	1|1	1|1	MS	HORVU1Hr1G086910
chr1H	538410620	A	T	0|0	0|0	1|1	1|1	3PU	HORVU1Hr1G086910
chr1H	538410632	TCTGA	T	0|0	0|0	1|1	1|1	3PU	HORVU1Hr1G086910
chr1H	538410649	G	T	0|0	0|0	1|1	1|1	3PU	HORVU1Hr1G086910
chr1H	538410664	A	G	0|0	0|0	1|1	1|1	3PU	HORVU1Hr1G086910
chr1H	538410669	C	T	0|0	0|0	1|1	1|1	SRI	HORVU1Hr1G086910
chr1H	538410768	G	A	0|0	0|0	1|1	1|1	3PU	HORVU1Hr1G086910
chr1H	538410805	C	T	0|0	0|0	1|1	1|1	3PU	HORVU1Hr1G086910
chr1H	538410842	A	C	0|0	0|0	1|1	1|1	3PU	HORVU1Hr1G086910
chr1H	538410904	G	A	0|0	0|0	1|1	1|1	MS	HORVU1Hr1G086910
chr1H	538410974	G	A	0|0	0|0	1|1	1|1	MS	HORVU1Hr1G086910
chr1H	538411002	G	A	0|0	0|0	1|1	1|1	MS	HORVU1Hr1G086910

chr1H	538411011	C	G	0|0	0|0	1|1	1|1	MS	HORVU1Hr1G086910
chr1H	538411030	C	G	0|0	0|0	1|1	1|1	MS	HORVU1Hr1G086910
chr1H	538411049	G	A	0|0	0|0	1|1	1|1	MS	HORVU1Hr1G086910
chr1H	538411161	G	A	0|0	0|0	1|1	1|1	SR	HORVU1Hr1G086910
chr1H	538411549	C	G	0|0	0|0	1|1	1|1	US	HORVU1Hr1G086920
chr1H	538411623	C	T	0|0	0|0	1|1	1|1	US	HORVU1Hr1G086920
chr1H	538411798	T	G	0|0	0|0	1|1	1|1	US	HORVU1Hr1G086920
chr1H	538411919	G	C	0|0	0|0	1|1	1|1	US	HORVU1Hr1G086920
chr1H	538411974	C	T	0|0	0|0	1|1	1|1	US	HORVU1Hr1G086920
chr1H	538411982	G	A	0|0	0|0	1|1	1|1	US	HORVU1Hr1G086920
chr1H	538412033	A	G	0|0	0|0	1|1	1|1	US	HORVU1Hr1G086920
chr1H	538412043	G	A	0|0	0|0	1|1	1|1	US	HORVU1Hr1G086920
chr1H	538412074	C	T	0|0	0|0	1|1	1|1	US	HORVU1Hr1G086920
chr1H	538412182	C	T	0|0	0|0	1|1	1|1	US	HORVU1Hr1G086920
chr1H	538412233	C	T	0|0	0|0	1|1	1|1	US	HORVU1Hr1G086920
chr1H	538412261	T	C	0|0	0|0	1|1	1|1	US	HORVU1Hr1G086920
chr1H	538412353	G	A	0|0	0|0	1|1	1|1	US	HORVU1Hr1G086920
chr1H	538412914	C	G	0|0	0|0	1|1	1|1	US	HORVU1Hr1G086920
chr1H	538412991	G	A	0|0	0|0	1|1	1|1	US	HORVU1Hr1G086920
chr1H	538413195	T	A	0|0	0|0	1|1	1|1	US	HORVU1Hr1G086920
chr1H	538413234	C	T	0|0	0|0	1|1	1|1	US	HORVU1Hr1G086920
chr1H	538415207	C	CGTTGCA	0|0	0|0	1|1	1|1	DII	HORVU1Hr1G086920
chr1H	538415273	G	A	0|0	0|0	1|1	1|1	MS	HORVU1Hr1G086920
chr1H	538415467	C	T	0|0	0|0	1|1	1|1	MS	HORVU1Hr1G086920
chr1H	538415715	C	A	0|0	0|0	1|1	1|1	MS	HORVU1Hr1G086920
chr1H	538419211	G	A	0|0	0|0	1|1	1|1	DG	HORVU1Hr1G086920
chr1H	538419891	G	A	0|0	0|0	1|1	1|1	DG	HORVU1Hr1G086920
chr1H	538420734	A	T	0|0	0|0	1|1	1|1	DG	HORVU1Hr1G086920
chr1H	538420777	G	T	0|0	0|0	1|1	1|1	DG	HORVU1Hr1G086920
chr1H	538421697	T	C	0|0	0|0	1|1	1|1	DG	HORVU1Hr1G086920
chr1H	538422174	G	C	0|0	0|0	1|1	1|1	DG	HORVU1Hr1G086930
chr1H	538423080	T	TG	0|0	0|0	1|1	1|1	3PU	HORVU1Hr1G086930
chr1H	538423409	G	A	0|0	0|0	1|1	1|1	SN	HORVU1Hr1G086930
chr1H	538423853	C	T	0|0	0|0	1|1	1|1	SN	HORVU1Hr1G086930
chr1H	538424409	T	C	0|0	0|0	1|1	1|1	5PU	HORVU1Hr1G086930
chr1H	538424798	G	A	0|0	0|0	1|1	1|1	5PUP	HORVU1Hr1G086930

chr1H	538424812	C	G	0|0	0|0	1|1	1|1	5PU	HORVU1Hr1G086930
chr1H	538426114	GA	G	0|0	0|0	1|1	1|1	5PU	HORVU1Hr1G086930
chr1H	538438400	C	T	0|0	0|0	1|1	1|1	IR	HORVU1Hr1G086930-HORVU1Hr1G086970
chr1H	538438408	C	A	0|0	0|0	1|1	1|1	IR	HORVU1Hr1G086930-HORVU1Hr1G086970
chr1H	538445011	C	A	0|0	0|0	1|1	1|1	IR	HORVU1Hr1G086930-HORVU1Hr1G086970
chr1H	538446130	T	C	0|0	0|0	1|1	1|1	IR	HORVU1Hr1G086930-HORVU1Hr1G086970
chr1H	538446693	G	A	0|0	0|0	1|1	1|1	IR	HORVU1Hr1G086930-HORVU1Hr1G086970
chr1H	538462245	G	T	0|0	0|0	1|1	1|1	IR	HORVU1Hr1G086930-HORVU1Hr1G086970
chr1H	538462522	G	C	0|0	0|0	1|1	1|1	IR	HORVU1Hr1G086930-HORVU1Hr1G086970
chr1H	538463214	G	T	0|0	0|0	1|1	1|1	IR	HORVU1Hr1G086930-HORVU1Hr1G086970
chr1H	538463867	C	T	0|0	0|0	1|1	1|1	IR	HORVU1Hr1G086930-HORVU1Hr1G086970
chr1H	538463900	C	A	0|0	0|0	1|1	1|1	IR	HORVU1Hr1G086930-HORVU1Hr1G086970
chr1H	538465774	A	G	0|0	0|0	1|1	1|1	IR	HORVU1Hr1G086930-HORVU1Hr1G086970
chr1H	538466031	A	C	0|0	0|0	1|1	1|1	IR	HORVU1Hr1G086930-HORVU1Hr1G086970
chr1H	538466332	G	C	0|0	0|0	1|1	1|1	IR	HORVU1Hr1G086930-HORVU1Hr1G086970
chr1H	538467537	C	T	0|0	0|0	1|1	1|1	IR	HORVU1Hr1G086930-HORVU1Hr1G086970
chr1H	538467702	C	CT	0|0	0|0	1|1	1|1	IR	HORVU1Hr1G086930-HORVU1Hr1G086970
chr1H	538468333	A	G	0|0	0|0	1|1	1|1	IR	HORVU1Hr1G086930-HORVU1Hr1G086970
chr1H	538468334	G	A	0|0	0|0	1|1	1|1	IR	HORVU1Hr1G086930-HORVU1Hr1G086970
chr1H	538468371	G	A	0|0	0|0	1|1	1|1	IR	HORVU1Hr1G086930-HORVU1Hr1G086970
chr1H	538468503	AG	A	0|0	0|0	1|1	1|1	IR	HORVU1Hr1G086930-HORVU1Hr1G086970
chr1H	538468900	G	A	0|0	0|0	1|1	1|1	IR	HORVU1Hr1G086930-HORVU1Hr1G086970
chr1H	538469144	GC	G	0|0	0|0	1|1	1|1	IR	HORVU1Hr1G086930-HORVU1Hr1G086970
chr1H	538470264	C	A	0|0	0|0	1|1	1|1	IR	HORVU1Hr1G086930-HORVU1Hr1G086970
chr1H	538470488	G	A	0|0	0|0	1|1	1|1	IR	HORVU1Hr1G086930-HORVU1Hr1G086970
chr1H	538470542	T	C	0|0	0|0	1|1	1|1	IR	HORVU1Hr1G086930-HORVU1Hr1G086970
chr1H	538470615	G	A	0|0	0|0	1|1	1|1	IR	HORVU1Hr1G086930-HORVU1Hr1G086970
chr1H	538470698	A	C	0|0	0|0	1|1	1|1	IR	HORVU1Hr1G086930-HORVU1Hr1G086970
chr1H	538470747	T	A	0|0	0|0	1|1	1|1	IR	HORVU1Hr1G086930-HORVU1Hr1G086970
chr1H	538470764	T	A	0|0	0|0	1|1	1|1	IR	HORVU1Hr1G086930-HORVU1Hr1G086970
chr1H	538470928	C	T	0|0	0|0	1|1	1|1	IR	HORVU1Hr1G086930-HORVU1Hr1G086970
chr1H	538471148	C	T	0|0	0|0	1|1	1|1	IR	HORVU1Hr1G086930-HORVU1Hr1G086970
chr1H	538471243	T	C	0|0	0|0	1|1	1|1	IR	HORVU1Hr1G086930-HORVU1Hr1G086970
chr1H	538471471	CA	C	0|0	0|0	1|1	1|1	IR	HORVU1Hr1G086930-HORVU1Hr1G086970
chr1H	538471716	C	T	0|0	0|0	1|1	1|1	IR	HORVU1Hr1G086930-HORVU1Hr1G086970
chr1H	538472081	C	T	0|0	0|0	1|1	1|1	IR	HORVU1Hr1G086930-HORVU1Hr1G086970

chr1H	538472110	C	T	0|0	0|0	1|1	1|1	IR	HORVU1Hr1G086930-HORVU1Hr1G086970
chr1H	538472281	G	A	0|0	0|0	1|1	1|1	IR	HORVU1Hr1G086930-HORVU1Hr1G086970
chr1H	538472449	T	C	0|0	0|0	1|1	1|1	IR	HORVU1Hr1G086930-HORVU1Hr1G086970
chr1H	538472545	G	A	0|0	0|0	1|1	1|1	IR	HORVU1Hr1G086930-HORVU1Hr1G086970
chr1H	538472563	C	T	0|0	0|0	1|1	1|1	IR	HORVU1Hr1G086930-HORVU1Hr1G086970
chr1H	538472592	G	A	0|0	0|0	1|1	1|1	IR	HORVU1Hr1G086930-HORVU1Hr1G086970
chr1H	538472597	G	A	0|0	0|0	1|1	1|1	IR	HORVU1Hr1G086930-HORVU1Hr1G086970
chr1H	538472616	G	A	0|0	0|0	1|1	1|1	IR	HORVU1Hr1G086930-HORVU1Hr1G086970
chr1H	538472674	G	C	0|0	0|0	1|1	1|1	IR	HORVU1Hr1G086930-HORVU1Hr1G086970
chr1H	538472695	G	A	0|0	0|0	1|1	1|1	IR	HORVU1Hr1G086930-HORVU1Hr1G086970
chr1H	538472782	C	T	0|0	0|0	1|1	1|1	IR	HORVU1Hr1G086930-HORVU1Hr1G086970
chr1H	538473078	C	T	0|0	0|0	1|1	1|1	IR	HORVU1Hr1G086930-HORVU1Hr1G086970
chr1H	538473176	A	G	0|0	0|0	1|1	1|1	IR	HORVU1Hr1G086930-HORVU1Hr1G086970
chr1H	538473659	C	T	0|0	0|0	1|1	1|1	IR	HORVU1Hr1G086930-HORVU1Hr1G086970
chr1H	538473907	C	T	0|0	0|0	1|1	1|1	IR	HORVU1Hr1G086930-HORVU1Hr1G086970
chr1H	538474131	G	GC	0|0	0|0	1|1	1|1	DG	HORVU1Hr1G086970
chr1H	538474238	G	T	0|0	0|0	1|1	1|1	DG	HORVU1Hr1G086970
chr1H	538474386	C	T	0|0	0|0	1|1	1|1	DG	HORVU1Hr1G086970
chr1H	538474464	C	T	0|0	0|0	1|1	1|1	DG	HORVU1Hr1G086970
chr1H	538474546	G	A	0|0	0|0	1|1	1|1	US	HORVU1Hr1G086980
chr1H	538474573	C	T	0|0	0|0	1|1	1|1	US	HORVU1Hr1G086980
chr1H	538474641	C	T	0|0	0|0	1|1	1|1	US	HORVU1Hr1G086980
chr1H	538474678	G	A	0|0	0|0	1|1	1|1	US	HORVU1Hr1G086980
chr1H	538474994	C	T	0|0	0|0	1|1	1|1	US	HORVU1Hr1G086980
chr1H	538475060	CGA	C	0|0	0|0	1|1	1|1	US	HORVU1Hr1G086980
chr1H	538475346	C	T	0|0	0|0	1|1	1|1	US	HORVU1Hr1G086980
chr1H	538475751	G	A	0|0	0|0	1|1	1|1	US	HORVU1Hr1G086980
chr1H	538478871	A	AAAG	0|0	0|0	1|1	1|1	US	HORVU1Hr1G086980
chr1H	538478892	G	T	0|0	0|0	1|1	1|1	US	HORVU1Hr1G086980
chr1H	538480604	A	G	0|0	0|0	1|1	1|1	5PU	HORVU1Hr1G086980
chr1H	538480835	T	C	0|0	0|0	1|1	1|1	5PU	HORVU1Hr1G086980
chr1H	538480963	G	C	0|0	0|0	1|1	1|1	5PU	HORVU1Hr1G086980
chr1H	538481017	G	C	0|0	0|0	1|1	1|1	5PU	HORVU1Hr1G086980
chr1H	538481189	G	A	0|0	0|0	1|1	1|1	5PU	HORVU1Hr1G086980
chr1H	538481338	A	G	0|0	0|0	1|1	1|1	5PU	HORVU1Hr1G086980
chr1H	538481648	C	T	0|0	0|0	1|1	1|1	5PU	HORVU1Hr1G086980

chr1H	538481658	A	T	0|0	0|0	1|1	1|1	5PU	HORVU1Hr1G086980
chr1H	538481659	C	T	0|0	0|0	1|1	1|1	5PU	HORVU1Hr1G086980
chr1H	538481772	A	AAAAAT	0|0	0|0	1|1	1|1	5PU	HORVU1Hr1G086980
chr1H	538481970	A	G	0|0	0|0	1|1	1|1	MS	HORVU1Hr1G086970
chr1H	538481993	G	A	0|0	0|0	1|1	1|1	SN	HORVU1Hr1G086970
chr1H	538482001	G	T	0|0	0|0	1|1	1|1	MS	HORVU1Hr1G086970
chr1H	538482316	C	T	0|0	0|0	1|1	1|1	MS	HORVU1Hr1G086980
chr1H	538483332	C	A	0|0	0|0	1|1	1|1	US	HORVU1Hr1G086970
chr1H	538483563	G	A	0|0	0|0	1|1	1|1	US	HORVU1Hr1G086970
chr1H	538484925	A	G	0|0	0|0	1|1	1|1	US	HORVU1Hr1G086970
chr1H	538485149	A	G	0|0	0|0	1|1	1|1	US	HORVU1Hr1G086970
chr1H	538486535	C	T	0|0	0|0	1|1	1|1	US	HORVU1Hr1G086970
chr1H	538487241	G	A	0|0	0|0	1|1	1|1	US	HORVU1Hr1G086970
chr1H	538487658	G	A	0|0	0|0	1|1	1|1	DG	HORVU1Hr1G086980
chr1H	538488524	C	T	0|0	0|0	1|1	1|1	DG	HORVU1Hr1G086980
chr1H	538489910	G	A	0|0	0|0	1|1	1|1	DG	HORVU1Hr1G086980
chr1H	538490049	C	A	0|0	0|0	1|1	1|1	DG	HORVU1Hr1G086980
chr1H	538490544	G	A	0|0	0|0	1|1	1|1	DG	HORVU1Hr1G086980
chr1H	538490611	A	T	0|0	0|0	1|1	1|1	DG	HORVU1Hr1G086980
chr1H	538490634	G	A	0|0	0|0	1|1	1|1	DG	HORVU1Hr1G086980
chr1H	538500001	T	A	0|0	0|0	1|1	1|1	US	HORVU1Hr1G087010
chr1H	538500020	G	C	0|0	0|0	1|1	1|1	US	HORVU1Hr1G087010
chr1H	538500133	G	A	0|0	0|0	1|1	1|1	US	HORVU1Hr1G087010
chr1H	538500300	G	A	0|0	0|0	1|1	1|1	US	HORVU1Hr1G087010
chr1H	538500523	A	G	0|0	0|0	1|1	1|1	US	HORVU1Hr1G087010
chr1H	538501926	C	T	0|0	0|0	1|1	1|1	US	HORVU1Hr1G087010
chr1H	538501941	G	A	0|0	0|0	1|1	1|1	US	HORVU1Hr1G087010
chr1H	538502493	G	C	0|0	0|0	1|1	1|1	US	HORVU1Hr1G087010
chr1H	538502692	C	T	0|0	0|0	1|1	1|1	SN	HORVU1Hr1G087010
chr1H	538502737	T	A	0|0	0|0	1|1	1|1	SRI	HORVU1Hr1G087010
chr1H	538502748	T	C	0|0	0|0	1|1	1|1	5PU	HORVU1Hr1G087010
chr1H	538502755	C	T	0|0	0|0	1|1	1|1	5PU	HORVU1Hr1G087010
chr1H	538502773	A	ACCAATAGAT	0|0	0|0	1|1	1|1	5PU	HORVU1Hr1G087010
chr1H	538502899	GC	G	0|0	0|0	1|1	1|1	US	HORVU1Hr1G087010
chr1H	538502914	TCATG	T	0|0	0|0	1|1	1|1	US	HORVU1Hr1G087010
chr1H	538502951	T	A	0|0	0|0	1|1	1|1	US	HORVU1Hr1G087010

chr1H	538502956	G	A	0|0	0|0	1|1	1|1	US	HORVU1Hr1G087010
chr1H	538502975	C	A	0|0	0|0	1|1	1|1	US	HORVU1Hr1G087010
chr1H	538502998	C	A	0|0	0|0	1|1	1|1	US	HORVU1Hr1G087010
chr1H	538503064	C	T	0|0	0|0	1|1	1|1	SN	HORVU1Hr1G087010
chr1H	538503228	T	C	0|0	0|0	1|1	1|1	5PU	HORVU1Hr1G087010
chr1H	538503234	T	A	0|0	0|0	1|1	1|1	5PU	HORVU1Hr1G087010
chr1H	538503235	C	A	0|0	0|0	1|1	1|1	5PU	HORVU1Hr1G087010
chr1H	538503251	C	T	0|0	0|0	1|1	1|1	5PUP	HORVU1Hr1G087010
chr1H	538503255	A	T	0|0	0|0	1|1	1|1	5PUP	HORVU1Hr1G087010
chr1H	538503298	G	A	0|0	0|0	1|1	1|1	SN	HORVU1Hr1G087010
chr1H	538503455	A	G	0|0	0|0	1|1	1|1	MS	HORVU1Hr1G087010
chr1H	538503456	C	A	0|0	0|0	1|1	1|1	MS	HORVU1Hr1G087010
chr1H	538503457	C	A	0|0	0|0	1|1	1|1	SN	HORVU1Hr1G087010
chr1H	538503518	T	C	0|0	0|0	1|1	1|1	SRI	HORVU1Hr1G087010
chr1H	538503519	A	G	0|0	0|0	1|1	1|1	SRI	HORVU1Hr1G087010
chr1H	538503520	G	C	0|0	0|0	1|1	1|1	SRI	HORVU1Hr1G087010
chr1H	538503535	C	T	0|0	0|0	1|1	1|1	5PU	HORVU1Hr1G087010
chr1H	538503552	GATGA	G	0|0	0|0	1|1	1|1	5PU	HORVU1Hr1G087010
chr1H	538503559	C	CCAGCAG	0|0	0|0	1|1	1|1	5PU	HORVU1Hr1G087010
chr1H	538503584	G	A	0|0	0|0	1|1	1|1	5PU	HORVU1Hr1G087010
chr1H	538503654	C	T	0|0	0|0	1|1	1|1	MS	HORVU1Hr1G087010
chr1H	538503832	A	C	0|0	0|0	1|1	1|1	MS	HORVU1Hr1G087010
chr1H	538505674	C	T	0|0	0|0	1|1	1|1	3PU	HORVU1Hr1G087010
chr1H	538506241	A	G	0|0	0|0	1|1	1|1	DG	HORVU1Hr1G087010
chr1H	538506738	G	A	0|0	0|0	1|1	1|1	DG	HORVU1Hr1G087010
chr1H	538507388	C	G	0|0	0|0	1|1	1|1	DG	HORVU1Hr1G087010
chr1H	538507437	G	T	0|0	0|0	1|1	1|1	DG	HORVU1Hr1G087010
chr1H	538507460	T	A	0|0	0|0	1|1	1|1	DG	HORVU1Hr1G087010
chr1H	538516556	G	T	0|0	0|0	1|1	1|1	IR	HORVU1Hr1G087020-HORVU1Hr1G087040
chr1H	538517671	AC	A	0|0	0|0	1|1	1|1	IR	HORVU1Hr1G087020-HORVU1Hr1G087040
chr1H	538523448	G	T	0|0	0|0	1|1	1|1	IR	HORVU1Hr1G087020-HORVU1Hr1G087040
chr1H	538545631	C	T	0|0	0|0	1|1	1|1	IR	HORVU1Hr1G087020-HORVU1Hr1G087040
chr1H	538546605	C	G	0|0	0|0	1|1	1|1	IR	HORVU1Hr1G087020-HORVU1Hr1G087040
chr1H	538553354	T	C	0|0	0|0	1|1	1|1	US	HORVU1Hr1G087040
chr1H	538595827	G	A	0|0	0|0	1|1	1|1	IR	HORVU1Hr1G087050-HORVU1Hr1G087070
chr1H	538597539	T	C	0|0	0|0	1|1	1|1	IR	HORVU1Hr1G087050-HORVU1Hr1G087070

chr1H	538600822	T	C	0|0	0|0	1|1	1|1	IR	HORVU1Hr1G087050-HORVU1Hr1G087070
chr1H	538601616	C	T	0|0	0|0	1|1	1|1	IR	HORVU1Hr1G087050-HORVU1Hr1G087070
chr1H	538601762	C	A	0|0	0|0	1|1	1|1	IR	HORVU1Hr1G087050-HORVU1Hr1G087070
chr1H	538602094	T	G	0|0	0|0	1|1	1|1	IR	HORVU1Hr1G087050-HORVU1Hr1G087070
chr1H	538602118	C	T	0|0	0|0	1|1	1|1	IR	HORVU1Hr1G087050-HORVU1Hr1G087070
chr1H	538602272	G	A	0|0	0|0	1|1	1|1	IR	HORVU1Hr1G087050-HORVU1Hr1G087070
chr1H	538602485	G	A	0|0	0|0	1|1	1|1	IR	HORVU1Hr1G087050-HORVU1Hr1G087070
chr1H	538602637	T	G	0|0	0|0	1|1	1|1	IR	HORVU1Hr1G087050-HORVU1Hr1G087070
chr1H	538602638	C	T	0|0	0|0	1|1	1|1	IR	HORVU1Hr1G087050-HORVU1Hr1G087070
chr1H	538603771	A	T	0|0	0|0	1|1	1|1	IR	HORVU1Hr1G087050-HORVU1Hr1G087070
chr1H	538605286	T	A	0|0	0|0	1|1	1|1	IR	HORVU1Hr1G087050-HORVU1Hr1G087070
chr1H	538621880	C	T	0|0	0|0	1|1	1|1	IR	HORVU1Hr1G087050-HORVU1Hr1G087070
chr1H	538632528	G	A	0|0	0|0	1|1	1|1	IR	HORVU1Hr1G087050-HORVU1Hr1G087070
chr1H	538632540	C	T	0|0	0|0	1|1	1|1	IR	HORVU1Hr1G087050-HORVU1Hr1G087070
chr1H	538690696	C	T	0|0	0|0	1|1	1|1	5PUP	HORVU1Hr1G087100
chr1H	538693200	G	T	0|0	0|0	1|1	1|1	MS	HORVU1Hr1G087100
chr1H	538693427	G	C	0|0	0|0	1|1	1|1	MS	HORVU1Hr1G087100
chr1H	538695929	TCA	T	0|0	0|0	1|1	1|1	US	HORVU1Hr1G087110
chr1H	538695948	C	T	0|0	0|0	1|1	1|1	US	HORVU1Hr1G087110
chr1H	538697209	G	T	0|0	0|0	1|1	1|1	US	HORVU1Hr1G087110
chr1H	538657757	C	CCGTCATA	0|0	0|0	1|1	1|1	US	HORVU1Hr1G087070
chr1H	538658317	CA	CAA;C	0|0	0|0	1|1	1|1	US	HORVU1Hr1G087070
chr1H	538660856	A	AT	0|0	0|0	1|1	1|1	3PU	HORVU1Hr1G087070
chr1H	538661549	CT	C	0|0	0|0	1|1	1|1	3PU	HORVU1Hr1G087070
chr1H	538661908	A	AGTTGC	0|0	0|0	1|1	1|1	DG	HORVU1Hr1G087070
chr1H	538662145	ATTAAGGATGGGGTGTACT	A	0|0	0|0	1|1	1|1	DG	HORVU1Hr1G087070
chr1H	538662163	TTTAAGGATGGGGTGTACTCC	T	0|0	0|0	1|1	1|1	DG	HORVU1Hr1G087070
chr1H	538662210	A	ACTAACTTTGTTTGAT	0|0	0|0	1|1	1|1	DG	HORVU1Hr1G087070
chr1H	538664716	GT;G	GTT	0|0	0|0	1|1	1|1	DG	HORVU1Hr1G087070
chr1H	538664774	CA	C	0|0	0|0	1|1	1|1	DG	HORVU1Hr1G087070
chr1H	538664980	C	CA	0|0	0|0	1|1	1|1	DG	HORVU1Hr1G087070
chr1H	538665486	T	TG	0|0	0|0	1|1	1|1	DG	HORVU1Hr1G087070
chr1H	538656272	A	G	0|0	0|0	1|1	1|1	US	HORVU1Hr1G087070
chr1H	538656772	T	A	0|0	0|0	1|1	1|1	US	HORVU1Hr1G087070
chr1H	538656870	C	T	0|0	0|0	1|1	1|1	US	HORVU1Hr1G087070
chr1H	538656978	T	C	0|0	0|0	1|1	1|1	US	HORVU1Hr1G087070

chr1H	538658121	A	G	0|0	0|0	1|1	1|1	US	HORVU1Hr1G087070
chr1H	538658260	A	G	0|0	0|0	1|1	1|1	US	HORVU1Hr1G087070
chr1H	538659583	C	T	0|0	0|0	1|1	1|1	SRI	HORVU1Hr1G087070
chr1H	538660204	G	A	0|0	0|0	1|1	1|1	3PU	HORVU1Hr1G087070
chr1H	538660730	A	G	0|0	0|0	1|1	1|1	3PU	HORVU1Hr1G087070
chr1H	538660885	T	C	0|0	0|0	1|1	1|1	3PU	HORVU1Hr1G087070
chr1H	538661314	G	T	0|0	0|0	1|1	1|1	SN	HORVU1Hr1G087070
chr1H	538661425	T	C	0|0	0|0	1|1	1|1	SN	HORVU1Hr1G087070
chr1H	538661473	T	C	0|0	0|0	1|1	1|1	SN	HORVU1Hr1G087070
chr1H	538661618	C	G	0|0	0|0	1|1	1|1	3PU	HORVU1Hr1G087070
chr1H	538661777	G	T	0|0	0|0	1|1	1|1	3PU	HORVU1Hr1G087070
chr1H	538662119	C	T	0|0	0|0	1|1	1|1	DG	HORVU1Hr1G087070
chr1H	538662234	T	C	0|0	0|0	1|1	1|1	DG	HORVU1Hr1G087070
chr1H	538662383	C	T	0|0	0|0	1|1	1|1	DG	HORVU1Hr1G087070
chr1H	538662440	A	G	0|0	0|0	1|1	1|1	DG	HORVU1Hr1G087070
chr1H	538662507	G	C	0|0	0|0	1|1	1|1	DG	HORVU1Hr1G087070
chr1H	538662526	T	C	0|0	0|0	1|1	1|1	DG	HORVU1Hr1G087070
chr1H	538662540	G	T	0|0	0|0	1|1	1|1	DG	HORVU1Hr1G087070
chr1H	538662608	T	C	0|0	0|0	1|1	1|1	DG	HORVU1Hr1G087070
chr1H	538662663	T	C	0|0	0|0	1|1	1|1	DG	HORVU1Hr1G087070
chr1H	538662767	T	C	0|0	0|0	1|1	1|1	DG	HORVU1Hr1G087070
chr1H	538662820	A	G	0|0	0|0	1|1	1|1	DG	HORVU1Hr1G087070
chr1H	538662843	C	T	0|0	0|0	1|1	1|1	DG	HORVU1Hr1G087070
chr1H	538662884	C	G	0|0	0|0	1|1	1|1	DG	HORVU1Hr1G087070
chr1H	538662950	C	A	0|0	0|0	1|1	1|1	DG	HORVU1Hr1G087070
chr1H	538663002	T	C	0|0	0|0	1|1	1|1	DG	HORVU1Hr1G087070
chr1H	538663089	C	T	0|0	0|0	1|1	1|1	DG	HORVU1Hr1G087070
chr1H	538663179	T	C	0|0	0|0	1|1	1|1	DG	HORVU1Hr1G087070
chr1H	538663233	A	T	0|0	0|0	1|1	1|1	DG	HORVU1Hr1G087070
chr1H	538663321	T	C	0|0	0|0	1|1	1|1	DG	HORVU1Hr1G087070
chr1H	538663410	A/T	G	0|0	0|0	1|1	1|1	DG	HORVU1Hr1G087070
chr1H	538663541	T	C	0|0	0|0	1|1	1|1	DG	HORVU1Hr1G087070
chr1H	538663603	T	C	0|0	0|0	1|1	1|1	DG	HORVU1Hr1G087070
chr1H	538663682	T	C	0|0	0|0	1|1	1|1	DG	HORVU1Hr1G087070
chr1H	538663919	T	C	0|0	0|0	1|1	1|1	DG	HORVU1Hr1G087070
chr1H	538664140	T	C	0|0	0|0	1|1	1|1	DG	HORVU1Hr1G087070

chr1H	538664184	T	C	0|0	0|0	1|1	1|1	DG	HORVU1Hr1G087070
chr1H	538664217	A	G	0|0	0|0	1|1	1|1	DG	HORVU1Hr1G087070
chr1H	538664347	A	G	0|0	0|0	1|1	1|1	DG	HORVU1Hr1G087070
chr1H	538664356	T	C	0|0	0|0	1|1	1|1	DG	HORVU1Hr1G087070
chr1H	538665031	A	C	0|0	0|0	1|1	1|1	DG	HORVU1Hr1G087070
chr1H	538665049	G	A	0|0	0|0	1|1	1|1	DG	HORVU1Hr1G087070
chr1H	538665526	C	A	0|0	0|0	1|1	1|1	DG	HORVU1Hr1G087070
chr1H	538665659	T	C	0|0	0|0	1|1	1|1	DG	HORVU1Hr1G087070
chr1H	538666342	G	T	0|0	0|0	1|1	1|1	DG	HORVU1Hr1G087070
chr1H	538666674	G	A	0|0	0|0	1|1	1|1	DG	HORVU1Hr1G087070
chr1H	538666764	C	T	0|0	0|0	1|1	1|1	DG	HORVU1Hr1G087070

Additional file 4. List of barley accessions used for genotype-based clustering analyses and genotype results.

Genotype data: "-" - no result; 1,2,3,4 represent different alleles according to their PCR product size ranking

				**Physicial position of markers**	523904076	533477747	536694265	537497331	537885088	538215819	538215819	538215819	538619248	538789299	538619248	539042699	544014147
**Accession ID**	**Possible origin**	**Origin Abreviation**	**Seed colour**	**Annotation**	**MC_2547043**	**MC_2546679**	**MC_45017**	**MC_53289**	**MC_1570156**	**MC_42987**	**MC_48835**	**MC_48835**	**MC_2550456**	**MC_162350**	**MC_48271**	**MC_1576759**	**MC_1578216**
00051_Eg	Egypt	Eg	Yellow		1	2	2	2	1	1	1	1	2	1	3	1	1
00071_Gr	Greece	Gr	Yellow		1	2	2	2	1	1	1	1	2	1	3	23	1
00296_Is	Israel	Is	Yellow		1	2	4	1	1	2	2	1	2	2	1	1	2
00298_In	India	In	Yellow		2	1	1	2	1	1	2	1	2	1	2	1	2
00317_UK	UK	UK	Yellow		1	2	3	2	1	1	1	1	2	1	3	23	1
00318_Is	Israel	Is	Yellow		1	2	4	-	1	2	2	1	2	2	1	1	2
00319_Is	Israel	Is	Yellow		1	2	1	2	1	1	1	1	2	1	3	1	1
00320_Is	Israel	Is	Yellow		1	1	-	2	1	2	2	1	2	1	1	12	1
00322_Cy	Cyperus	Cy	Yellow		1	2	3	-	1	1	1	1	2	1	2	1	1
00345_Ch	China	Ch	Yellow		2	2	1	2	1	1	1	1	1	1	2	1	2
00473_UK	UK	UK	Yellow		2	2	4	1	1	2	2	1	2	2	1	1	2
00909_Et	Ethiopia	Et	Yellow		1	2	3	2	1	1	1	1	2	1	3	3	1
02341_Ch	China	Ch	Yellow		1	1	2	2	1	1	1	23	2	1	2	1	1
02346_Et	Ethiopia	Et	Yellow		1	12	2	2	12	1	1	23	2	1	23	1	1
02348_Ch	China	Ch	Yellow		1	2	2	2	1	1	1	1	2	1	3	1	1
02352_Ch	China	Ch	Yellow		2	2	1	2	1	1	2	1	2	1	2	1	1
02354_Ch	China	Ch	Yellow		1	2	2	2	1	2	2	1	2	2	1	3	1
02371_So	South Korea	So	Yellow		2	2	1	2	1	1	2	1	2	1	2	1	2
02394_Et	Ethiopia	Et	Yellow		1	2	1	2	1	1	1	23	2	1	2	1	2
02564_Mo	Morocco	Mo	Yellow		1	2	4	1	1	2	2	1	2	2	1	1	2
02594_Sy	Syria	Sy	Black		1	1	3	2	2	1	1	3	-	2	2	1	1
02597_Sy	Syria	Sy	Black		1	1	3	2	1	1	1	3	1	2	4	1	1
02600_Sy	Syria	Sy	Yellow		1	1	3	2	2	1	1	3	2	2	2	1	1
02613_Sy	Syria	Sy	Black		1	1	2	-	1	-	-	3	-	2	4	1	1
02617_Sy	Syria	Sy	Black		1	1	3	2	1	1	1	3	1	1	4	1	1
02618_Sy	Syria	Sy	Black		1	1	3	2	1	1	1	3	1	2	4	1	1
02622_Sy	Syria	Sy	Black		1	1	3	2	1	1	1	3	1	2	4	1	1
02623_Sy	Syria	Sy	Black		1	1	3	2	1	1	1	3	1	2	4	1	1
02626_Sy	Syria	Sy	Black		1	1	-	2	1	1	1	3	1	2	4	1	1
02627_Sy	Syria	Sy	Black		1	1	3	2	1	1	1	3	1	2	4	1	2
02631_Sy	Syria	Sy	Black		1	1	1	2	1	1	1	3	1	2	4	1	1
02632_Sy	Syria	Sy	Black		1	1	2	2	1	1	1	3	1	2	4	1	1
02636_Sy	Syria	Sy	Black		1	1	2	2	1	1	1	3	1	2	4	1	1
02637_Sy	Syria	Sy	Black		1	1	3	2	1	2	1	3	1	2	4	1	1
02638_Sy	Syria	Sy	Black		1	1	2	2	1	1	1	3	2	2	2	1	1
02639_Sy	Syria	Sy	Black		1	1	2	2	1	1	1	3	1	2	4	1	1
02640_Sy	Syria	Sy	Black		1	1	2	2	1	1	1	3	1	2	4	1	1
02642_Sy	Syria	Sy	Black		1	1	2	2	1	1	1	3	1	2	4	1	1
02646_Sy	Syria	Sy	Black		1	1	2	1	1	1	1	3	1	2	-	1	-
02668_Sy	Syria	Sy	Yellow		1	1	2	2	1	1	1	3	-	2	2	1	1
02674_Sy	Syria	Sy	Yellow		1	1	3	2	1	1	1	3	1	2	4	1	1

02677_Sy	Syria	Sy	Yellow		1	1	3	2	1	1	1	23	-	1	3	12	1
02680_Sy	Syria	Sy	Yellow	Wild	1	2	1	1	1	1	1	3	1	-	4	1	-
02861_Tu	Turkey	Tu	Yellow		1	2	1	2	1	1	12	1	2	12	23	13	1
02862_Tu	Turkey	Tu	Yellow		1	1	3	2	1	2	2	1	2	1	1	1	1
02867_Al	Algerian	Al	Yellow		1	1	1	2	2	2	2	1	1	1	2	1	1
02881_Eg	Egypt	Eg	Yellow		1	2	3	2	1	1	1	1	2	1	3	3	1
02882_Bo	Bolivian	Bo	Yellow		1	2	3	2	1	1	1	1	2	2	3	3	1
02899_Cz	Czech	Cz	Yellow		2	1	1	2	2	2	2	1	1	1	2	1	1
03035_Ch	China	Ch	Yellow		1	1	1	2	2	2	2	1	1	1	2	1	1
03083_Et	Ethiopia	Et	Yellow		1	1	2	2	1	1	1	23	2	1	2	1	1
03257_US	USA	US	Yellow		1	2	3	2	1	1	1	1	1	1	2	1	1
03258_US	USA	US	Yellow		1	2	4	1	1	1	1	1	2	2	3	3	1
03399_Tu	Turkey	Tu	Yellow		1	2	3	2	1	1	1	1	2	2	3	3	1
03441_Eg	Egypt	Eg	Yellow		1	2	2	2	1	12	12	1	2	1	13	123	1
03447_Tu	Turkey	Tu	Yellow		1	1	3	2	1	2	2	1	2	2	1	1	1
03920_Tu	Turkey	Tu	Yellow		1	1	4	1	1	1	1	1	2	2	3	3	1
04041_Mo	Morocco	Mo	Yellow		1	1	4	1	1	1	1	1	2	2	2	3	1
04814_Ir	Iran	Ir	Yellow		1	2	4	1	1	2	2	1	2	2	1	1	2
04874_Ch	China	Ch	Yellow		1	2	1	2	1	1	2	1	2	1	2	1	2
06017_Ec	Ecuador	Ec	Yellow		1	1	2	2	1	1	1	23	2	1	2	1	1
06372_Eg	Egypt	Eg	Yellow		1	1	1	2	2	1	1	23	1	1	2	12	1
06381_Tu	Turkey	Tu	Yellow		1	1	3	2	1	2	2	1	2	2	1	1	1
06383_Af	Afghanistan	Af	Yellow		1	2	1	2	1	1	2	1	2	1	2	1	2
06540_US	USA	US	Yellow		1	2	4	1	1	2	2	1	2	2	1	1	2
06545_So	South Korea	So	Yellow		2	2	1	2	1	1	2	1	2	1	2	1	2
06553_Et	Ethiopia	Et	Yellow		1	1	1	2	1	1	1	23	2	1	2	1	2
06557_Et	Ethiopia	Et	Yellow		1	2	1	2	1	1	1	23	2	1	2	1	2
06563_Ch	China	Ch	Yellow		12	2	14	12	1	12	12	1	12	12	12	1	2
06603_Et	Ethiopia	Et	Yellow		1	2	1	2	1	1	1	23	2	1	2	1	1
06657_Sp	Spain	Sp	Yellow		1	2	2	2	2	1	1	23	2	2	1	1	1
06663_Et	Ethiopia	Et	Black		1	1	1	2	2	1	1	23	2	1	2	1	1
07112_Sy	Syria	Sy	Black		1	1	1	2	1	1	1	3	-	1	2	1	1
07147_Ja	Japan	Ja	Yellow		1	2	4	1	1	1	1	1	2	2	2	3	1
07386_Ja	Japan	Ja	Yellow		1	2	4	1	1	1	1	13	2	2	3	3	1
07389_Ja	Japan	Ja	Yellow		1	2	4	1	1	1	1	1	2	2	3	3	1
07401_Eg	Egypt	Eg	Yellow		1	1	4	12	1	12	12	1	2	12	23	13	1
07403_In	India	In	Yellow		2	1	1	2	1	1	2	1	1	1	2	1	2
07404_Az	Azerbaijan	Az	Yellow		1	1	3	2	1	1	1	23	-	1	3	1	1
07415_Al	Algeria	Al	Yellow		1	2	2	1	1	2	2	1	2	2	1	1	1
08655_Fr	France	Fr	Yellow		1	1	2	2	1	1	1	3	2	2	2	1	1
08746_Ru	Russia	Ru	Yelow		1	2	1	2	1	1	1	23	2	1	2	1	2
08778_Sy	Syria	Sy	Black		1	1	2	2	1	1	1	3	-	2	2	1	1
08864_Sy	Syria	Sy	Black		1	1	2	2	1	1	1	3	2	2	2	1	1
09717_Ch	China	Ch	Black		1	1	-	2	1	1	1	3	1	1	4	1	2
09769_Po	Portugal	Po	Yellow	Wild	1	-	1	2	1	12	-	13	1	-	2	1	1
09804_Po	Portugal	Po	Black		1	2	3	2	1	1	1	3	12	2	4	1	1

09836_Sp	Spain	Sp	Yellow		1	1	4	1	1	1	1	1	2	2	3	3	1
09837_Sp	Spain	Sp	Yellow		1	12	2	2	2	1	1	2	2	2	1	1	2
10528_Ch	China	Ch	Yellow		1	2	4	1	1	1	1	1	2	2	3	3	1
10576_Ch	China	Ch	Yellow		1	2	4	1	1	1	1	1	2	2	3	3	1
10595_Ch	China	Ch	Yellow		1	2	1	2	1	1	2	1	2	1	2	1	2
11723_Is	Israel, Lahav, Judean Foothills	Is	Yellow	Wild	1	1	-	2	1	1	1	23	2	1	3	1	2
11724_Eg	Egypt	Eg	Yellow		1	1	3	2	1	2	2	1	2	1	1	12	1
11727_Is	Israel	Is	Yellow	Wild	1	2	3	2	1	1	1	1	12	1	1	1	1
11728_Is	Israel, Hanita	Is	Yellow	Wild	1	2	1	2	1	2	2	1	1	2	2	1	1
11729_Is	Israel	Is	Yellow	Wild	1	2	2	2	1	1	1	23	2	2	3	1	1
11730_Is	Israel, Bat Shelomo	Is	Yellow	Wild	1	2	-	2	1	1	1	23	2	2	3	1	1
11731_Is	Israel	Is	Yellow	Wild	1	2	1	2	1	2	2	1	12	1	3	12	-
11732_Is	Isael	Is	Yellow	Wild	1	1	-	2	1	2	2	1	2	2	3	12	-
11733_Is	Israel, Sasa, Upper Galilee,	Is	Yellow	Wild	1	2	-	2	1	1	1	23	2	2	3	12	-
11736_Is	Israel	Is	Yellow	Wild	1	1	1	2	1	2	2	1	2	1	2	12	2
11737_Is	Israel	Is	Yellow	Wild	1	1	1	2	1	2	2	1	2	1	2	12	2
11738_Is	Israel	Is	Yellow	Wild	1	2	1	2	1	2	2	1	2	1	2	12	-
12140_Ir	Iraq	Ir	Yellow		1	1	3	2	1	1	2	1	2	1	2	1	1
12150_Ir	Iraq	Ir	Yellow		1	1	1	2	1	1	1	23	2	1	q	1	2
12151_Ir	Iraq	Ir	Black		1	1	1	2	12	12	12	13	1	1	q	1	1
12154_Ir	Iraq	Ir	Black		1	2	1	2	1	1	1	3	1	1	4	1	-
12159_Af	Afghanistan	Af	Yellow		1	2	1	2	1	1	2	1	2	1	2	1	1
12673_Ir	Iran	Ir	Yellow		1	1	3	2	1	12	12	1	2	1	13	1	1
12687_Jo	Jordan	Jo	Yellow		1	2	3	2	1	1	1	1	2	1	3	32	1
12688_Jo	Jordan	Jo	Yellow		1	2	1	2	1	1	1	1	2	2	3	3	1
12719_Pa	Pakistan	Pa	Yellow		1	1	1	2	1	2	2	1	2	1	1	1	1
12730_Ru	Russia	Ru	Yellow		1	1	3	2	1	2	2	1	1	1	2	1	1
12737_Ir	Iran	Ir	Yellow		1	2	1	2	1	1	2	1	2	-	3	1	2
12746_Jo	Jordan	Jo	Yellow		1	1	3	2	1	2	2	1	2	1	1	12	1
12748_Jo	Jordan	Jo	Yellow		1	1	1	2	1	2	2	1	1	1	3	23	1
12759_Et	Ethiopia	Et	Black		1	1	1	2	1	1	1	23	2	1	2	1	2
12763_Ir	Iraq	Ir	Yellow		1	1	1	2	1	1	2	1	2	1	2	1	-
12764_Ar	Armenia	Ar	Yellow		1	1	3	2	2	1	1	23	-	1	3	1	1
12794_Ir	Iraq	Ir	Black		1	1	3	2	1	1	1	3	1	1	-	1	1
12808_Ar	Armenia	Ar	Black		1	1	3	3	2	1	1	3	2	1	3	1	1
12830_Ge	Georgia	Ge	Black		1	1	1	2	1	1	1	3	2	1	3	1	1
12863_Uz	Uzbekistan	Uz	Yellow		1	2	3	2	1	2	2	1	2	2	1	1	1
12881_Az	Azerbaijan	Az	Black		1	1	3	3	1	1	1	3	2	1	3	1	1
12893_Az	Azerbaijan	Az	Black		1	1	3	3	1	1	1	3	2	1	3	1	1
12944_Ch	China	Ch	Black		1	1	3	2	1	1	1	3	1	2	4	1	2
12946_Ch	China	Ch	Black		2	1	3	2	1	1	1	3	1	2	4	1	2
13074_Ko	Korea	Ko	Yellow, Hulless		1	2	1	2	1	1	2	1	2	1	2	1	2

13357_Az	Azerbaijan	Az	Black		1	1	1	2	1	1	1	3	2	1	3	1	1
13360_Uz	Uzbekistan	Uz	Black		1	1	1	2	1	1	1	3	2	1	3	1	1
13363_Ar	Armenia	Ar	Yellow		1	1	3	2	1	1	1	23	2	1	3	1	2
13378_Ge	Georgia	Ge	Black		1	1	1	2	1	1	1	3	2	1	3	1	1
13397_Ir	Iraq	Ir	Yellow		1	2	1	2	1	1	2	1	2	1	2	1	-
13414_Uz	Uzbekistan	Uz	Yellow		1	2	3	2	1	2	2	1	2	1	1	1	1
13493_Ge	Georgia	Ge	Yellow		1	1	2	2	1	1	1	1	2	2	1	1	1
13503_Ka	Kazakhstan	Ka	Yellow		1	2	3	2	1	1	1	1	2	2	3	3	1
13509_Ka	Kazakhstan	Ka	Yellow		1	2	3	2	1	1	1	1	2	2	3	3	1
13510_Uz	Uzbekistan	Uz	Yellow		2	2	3	2	1	1	1	1	2	2	3	3	1
13514_Ka	Kazakhstan	Ka	Yellow		2	2	1	2	1	1	1	1	1	1	2	1	1
B1_Ch	China	Ch	Yellow		1	1	3	2	1	1	1	3	1	1	4	1	1
B1306_Ch	China	Ch	Yellow		2	2	2	2	1	1	2	1	2	1	2	1	2
B1308_Ch	China	Ch	Yellow		1	1	3	2	1	1	1	23	2	-	3	1	2
B1310_Ch	China	Ch	Yellow		2	1	2	2	1	1	2	1	2	1	23	1	12
B2_Ch	China	Ch	Yellow		1	2	2	2	1	1	1	2	2	1	2	1	1
DONGQING15	Tibet	Ti	Yellow, Hulless		1	1	3	2	1	1	2	1	1	1	2	1	2
DONGQING16	Tibet	Ti	Yellow, Hulless		1	1	3	2	1	1	2	1	1	1	2	1	2
EC1_NFS_03_I	Israel, North Facing Slope	Is	Yellow	Wild	1	2	2	2	1	1	1	1	1	1	1	1	-
EC1_NFS_04_I	Israel, North Facing Slope	Is	Yellow	Wild	1	2	2	2	1	1	1	1	1	1	1	1	-
EC1_NFS_05_I	Israel, North Facing Slope	Is	Yellow	Wild	1	2	2	2	1	1	1	1	1	1	1	1	-
EC1_NFS_06_I	Israel, North Facing Slope	Is	Yellow	Wild	1	2	2	2	1	2	2	1	-	1	3	1	-
EC1_NFS_07_I	Israel, North Facing Slope	Is	Yellow	Wild	1	2	2	2	1	2	2	1	-	1	3	1	-
EC1_NFS_08_I	Israel, North Facing Slope	Is	Yellow	Wild	1	2	-	2	1	1	1	123	1	1	2	123	2
EC1_NFS_09_I	Israel, North Facing Slope	Is	Yellow	Wild	1	-	2	2	1	1	1	1	1	1	1	1	-
EC1_NFS_10_I	Israel, North Facing Slope	Is	Yellow	Wild	1	2	2	2	1	2	2	1	-	1	3	1	-
EC1_NFS_11_I	Israel, North Facing Slope	Is	Yellow	Wild	1	2	2	2	1	2	2	1	-	1	3	1	-
EC1_SFS_12_I	Israel, South Facing Slope	Is	Yellow	Wild	1	2	2	2	1	2	2	1	-	1	3	1	-
EC1_SFS_13_I	Israel, South Facing Slope	Is	Yellow	Wild	1	2	2	2	1	2	2	1	-	1	3	1	-
EC1_SFS_14_I	Israel, South Facing Slope	Is	Yellow	Wild	1	2	2	2	1	2	2	1	2	1	2	1	-
EC1_SFS_15_I	Israel, South Facing Slope	Is	Yellow	Wild	1	2	2	2	1	2	2	1	2	1	2	1	-
EC1_SFS_16_I	Israel, South Facing Slope	Is	Yellow	Wild	1	2	2	2	1	2	-	13	2	1	2	1	-

EC1_SFS_17_I	Israel, South Facing Slope	Is	Yellow	Wild	1	2	2	2	1	2	2	1	-	1	2	1	-
Kino_Nijo_15_	Japan	Ja	Yellow		1	1	14	12	12	12	12	1	12	12	23	13	1
PuTian_WuDu	China	Ch	Purple		1	2	1	2	1	1	2	1	2	1	2	1	2
W1_Ti	Tibet	Ti	Black	Wild	2	1	3	2	1	1	1	3	1	1	4	1	2
W2_Ti	Tibet	Ti	Yellow	Wild	1	2	3	2	1	2	2	1	2	1	1	12	1
X1_Ti	Tibet	Ti	Black	Wild	1	2	2	-	1	1	-	3	1	1	4	1	2
XIMALA22_Ti	Tibet	Ti	Yellow		2	1	2	2	1	1	2	1	2	1	3	1	2
XZ46_Ti	Tibet	Ti	Black		1	2	4	2	1	1	1	3	12	12	4	13	1
XZ54_Ti	Tibet	Ti	Black		2	1	-	2	1	1	1	3	1	1	-	1	2
XZ76_Ti	Tibet	Ti	Black		2	1	3	2	1	1	1	3	1	1	4	1	2
YANGCIMAI3_	China	Ch	Yellow		1	2	4	1	1	1	1	1	2	2	3	3	1
Z_AU3_Ch	China	Ch	Yellow		1	2	4	1	1	1	1	1	2	2	2	3	1
ZANGQING23_	Tibet	Ti	Yellow, Hulless		1	1	2	2	1	1	2	1	2	1	3	1	2
ZANGQING25_	Tibet	Ti	Yellow, Hulless		1	1	2	2	1	1	2	1	2	1	3	1	2
ZANGQING31	Tibet	Ti	Yellow, Hulless		2	1	3	2	1	1	2	1	1	1	2	1	2
ZANGQING32	Tibet	Ti	Yellow, Hulless		1	1	2	-	1	1	2	1	2	1	3	1	2
ZANGQING69	Tibet	Ti	Yellow, Hulless		2	2	2	2	1	1	2	1	2	1	2	1	2

Additional file 5. Representative gel pictures for the markers used for genotyping analyses.

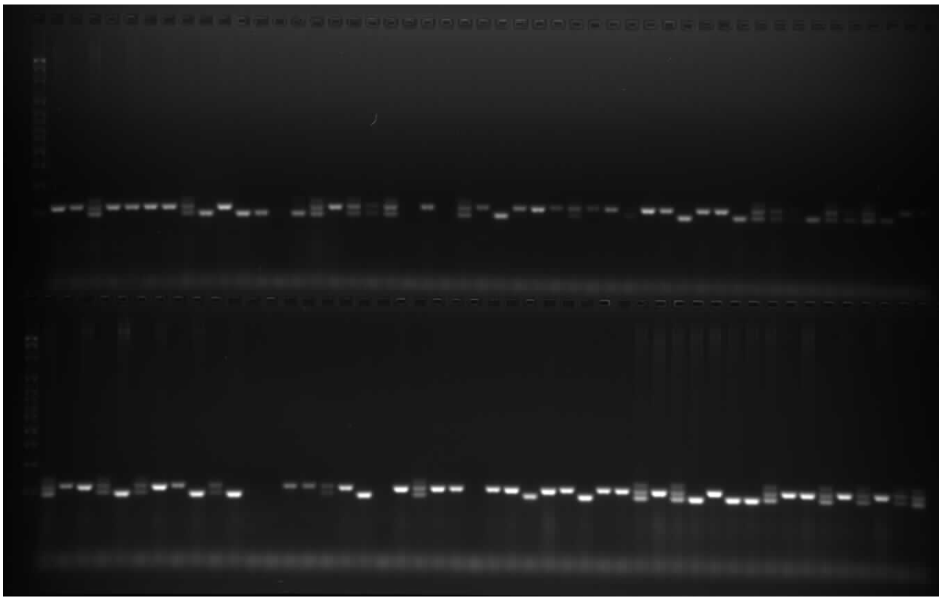

MC_2547043

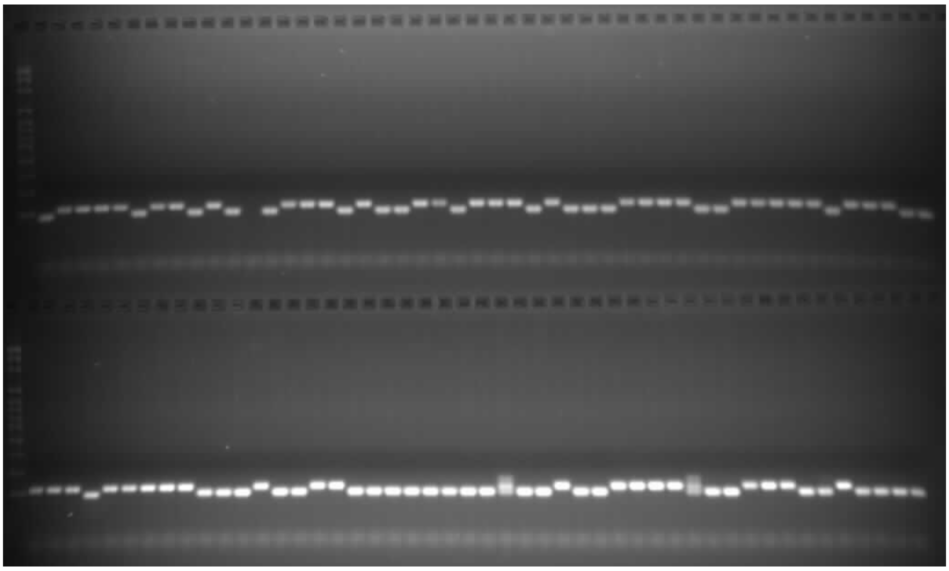

MC_2546679

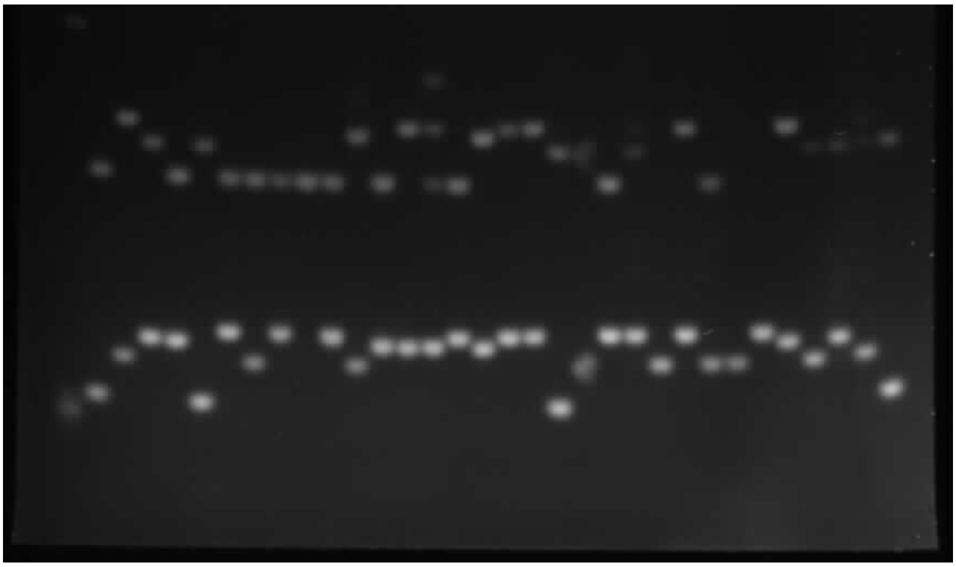

MC_45017 (4 alleles, double loading gel)

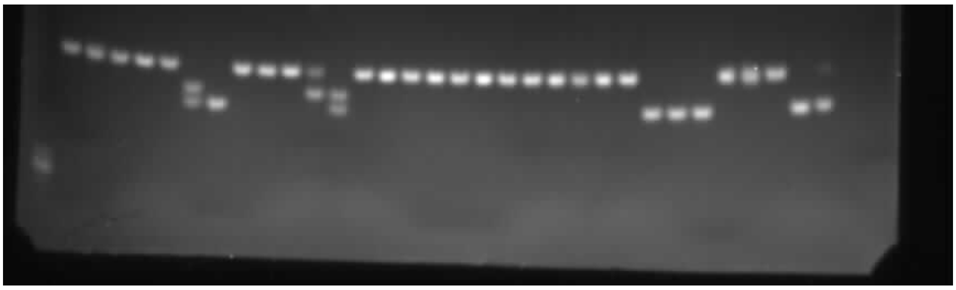

MC_53289 (3 alleles)

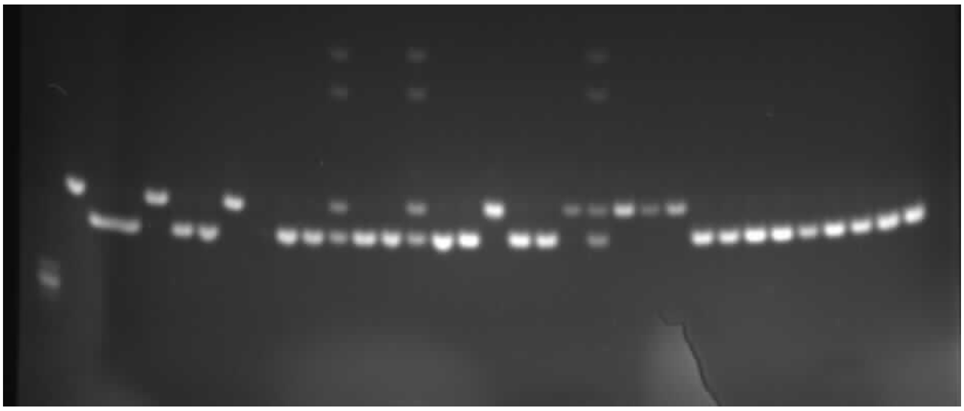

MC_1570156

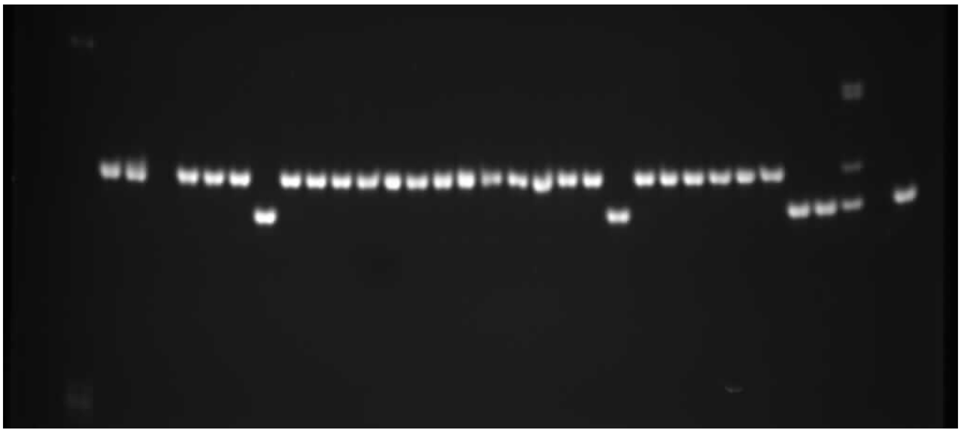

MC_42987

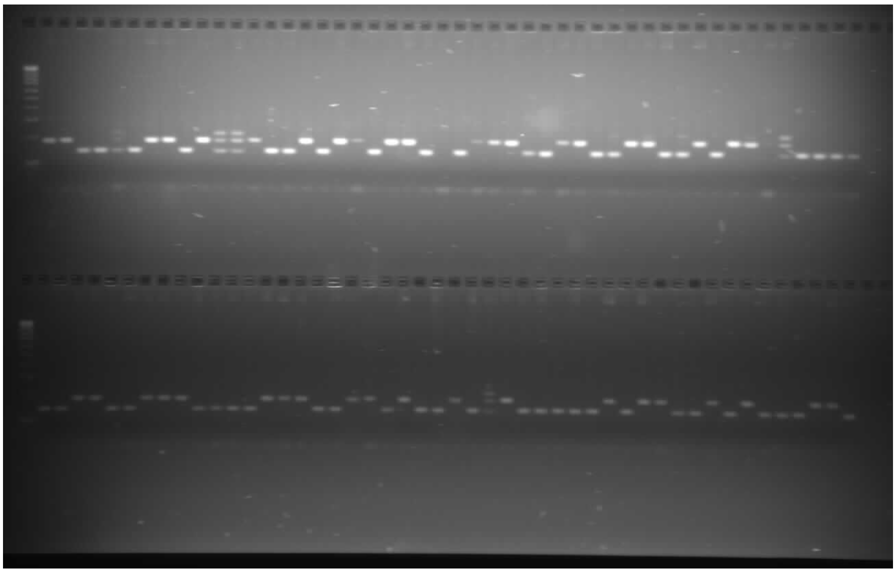

MC_488351

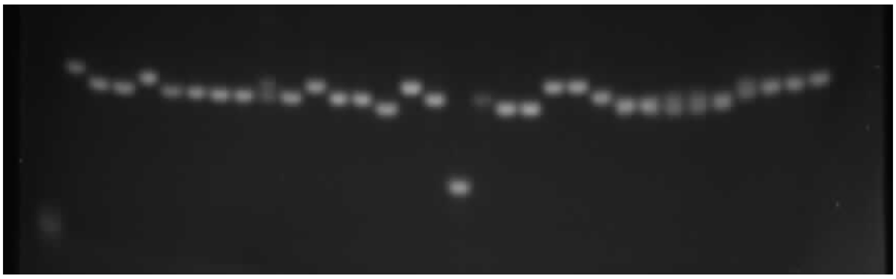

MC_488352 (3 alleles)

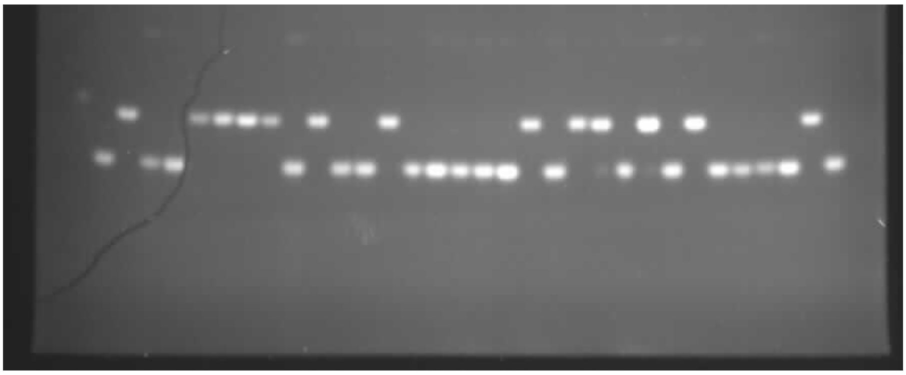

MC_2550456

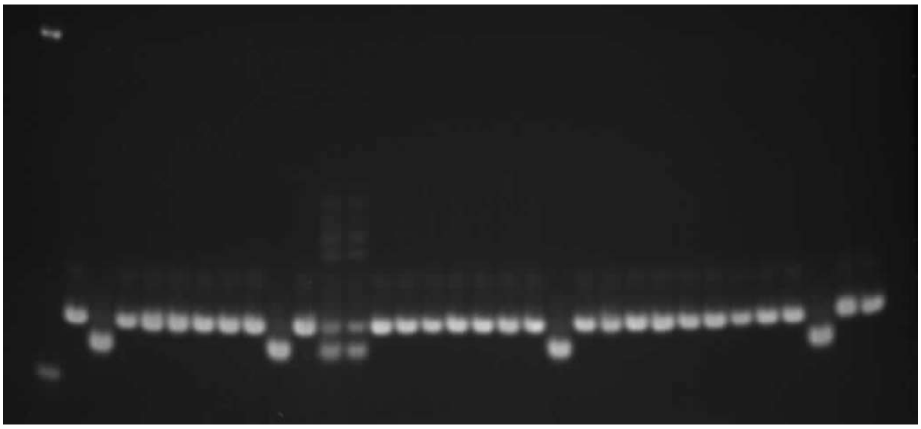

MC_162350

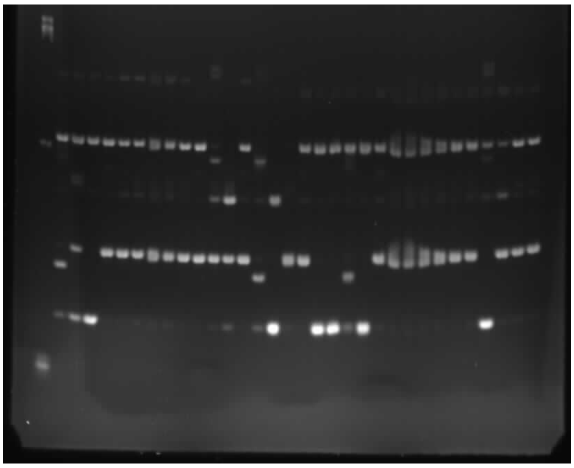

MC_48271 (3 alleles, double loading gel)

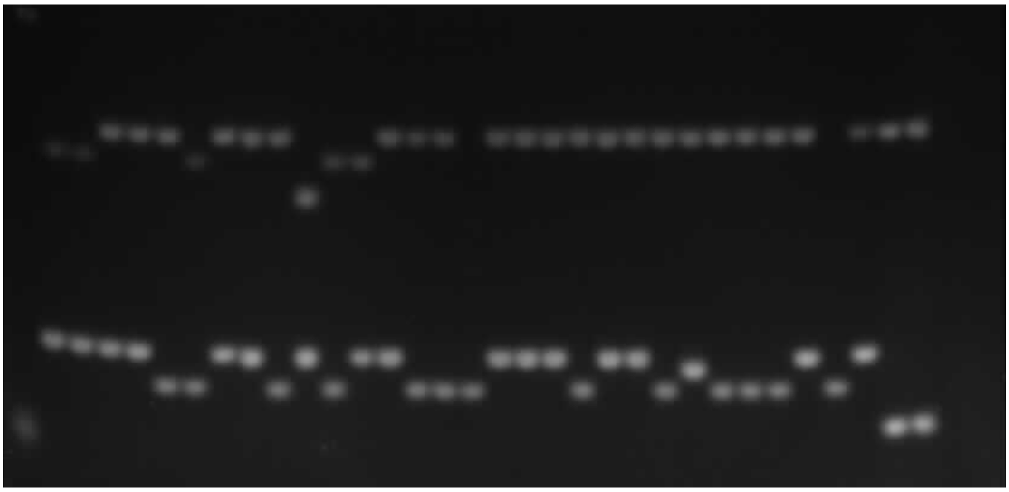

MC_1576759 (3 alleles, double loading gel)

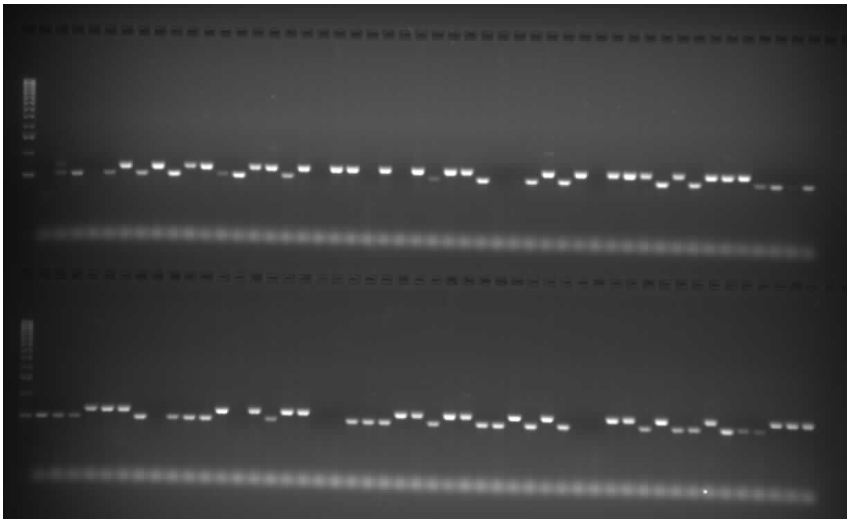

MC_1578216
